# Interaction between phenylpropane metabolism and oil accumulation in the developing seed of *Brassica napus* revealed by high temporal-resolution transcriptomes

**DOI:** 10.1186/s12915-023-01705-z

**Published:** 2023-09-29

**Authors:** Liangqian Yu, Dongxu Liu, Feifan Yin, Pugang Yu, Shaoping Lu, Yuting Zhang, Hu Zhao, Chaofu Lu, Xuan Yao, Cheng Dai, Qing-Yong Yang, Liang Guo

**Affiliations:** 1 National Key Laboratory of Crop Genetic Improvement, Hubei Hongshan Laboratory, Huazhong Agricultural University, Wuhan, 430070 China; 2 Hubei Key Laboratory of Agricultural Bioinformatics, College of Informatics, Huazhong Agricultural University, Wuhan, 430070 China; 3 Yazhouwan National Laboratory, Sanya, 572025 China; 4 Department of Plant Sciences and Plant Pathology, Montana State University, Bozeman, 59717 USA

**Keywords:** *Brassica napus*, Transcriptome, Co-expression, Seed development, Seed coat, Carbon partitioning, Oil biosynthesis

## Abstract

**Background:**

*Brassica napus* is an important oilseed crop providing high-quality vegetable oils for human consumption and non-food applications. However, the regulation between embryo and seed coat for the synthesis of oil and phenylpropanoid compounds remains largely unclear.

**Results:**

Here, we analyzed the transcriptomes in developing seeds at 2-day intervals from 14 days after flowering (DAF) to 64 DAF. The 26 high-resolution time-course transcriptomes are clearly clustered into five distinct groups from stage I to stage V. A total of 2217 genes including 136 transcription factors, are specifically expressed in the seed and show high temporal specificity by being expressed only at certain stages of seed development. Furthermore, we analyzed the co-expression networks during seed development, which mainly included master regulatory transcription factors, lipid, and phenylpropane metabolism genes. The results show that the phenylpropane pathway is prominent during seed development, and the key enzymes in the phenylpropane metabolic pathway, including *TT5*, *BAN*, and the transporter *TT19*, were directly or indirectly related to many key enzymes and transcription factors involved in oil accumulation. We identified candidate genes that may regulate seed oil content based on the co-expression network analysis combined with correlation analysis of the gene expression with seed oil content and seed coat content.

**Conclusions:**

Overall, these results reveal the transcriptional regulation between lipid and phenylpropane accumulation during *B. napus* seed development. The established co-expression networks and predicted key factors provide important resources for future studies to reveal the genetic control of oil accumulation in *B. napus* seeds.

**Supplementary Information:**

The online version contains supplementary material available at 10.1186/s12915-023-01705-z.

## Background

As an important oil crop, rapeseed (*Brassica napus*; AACC, 2*n* = 38) originates from a hybridization between *Brassica rapa* (AA, 2*n* = 20) and *Brassica oleracea* (CC, 2*n* = 18). Rapeseed oil is widely accepted for human consumption and as a raw material for industrial use [1]. An increasing evidence also supports rapeseed oil as a health-promoting dietary ingredient [2]. In recent years, the demand for vegetable oil has increased sharply, and improving oil yield and quality has become a major breeding goal for *B. napus*. One of the important traits is the seed color, which is determined by the deposition of pigments in the seed coat. Compared with black-seeded rapeseed, yellow-seeded rapeseed has the advantages of thin seed coat, low content of anti-nutritional factors, high content of protein and oil, and clear oil color [3–5], making yellow seed a favorable trait in *B. napus*. However, limited progress has been made in the regulatory mechanisms of oil accumulation in rapeseed and its coordination with the metabolism for seed coat development.

Seed development is a complex and dynamic process consisting of the development of the zygotic embryo and endosperm and the maternally derived seed coat [6]. Seed development can be roughly divided into three stages including embryogenesis, maturation, and desiccation. The process of seed development usually begins with cell division and tissue differentiation during embryonic development. Accumulation of storage reserves, mainly protein and oil in the form of triacylglycerol (TAG), happens in the maturation stage followed by a desiccation/quiescence phase to produce dry oilseeds [7]. Typically, *B. napus* seeds contain 40% oil and 15% protein [8]. As the major storage reserve in seeds, TAG is assembled in the endoplasmic reticulum (ER) under the action of glycerol-3-P acyltransferases using glycerol-3-P and fatty acids (FAs) originally synthesized in the plastid [9–11].

Seed coat serves as a protective function throughout development. It also releases nutrients and transmits signals from the maternal tissues to the embryo, which are necessary for proper embryo development [12]. The accumulation of seed coat-specific flavonoids, mainly the phenylpropane compounds cyanidin and procyanidins (PAs) [13, 14], is a characteristic step of seed maturation in many species [15]. The contents of these compounds determine the color of mature *B. napus* seed. Cyanidin and procyanidins, together with flavonols, phlobaphenes, and isoflavones, all belong to flavonoids that possess a common C6-C3-C6 body [16]. The seed coat contents of flavonol and procyanidin in the black-seeded varieties are higher than those of the yellow-seeded varieties [13]. In *B. napus*, the soluble procyanidin content increases throughout the seed development to reach a maximum level during the early seed maturation stage (30 day after pollination (DAF)) and then decreases dramatically and concomitantly with the onset of seed browning (from 40 DAF onward), followed by a maximum level of insoluble procyanidin accumulation [17]. The accumulation of these flavonoids in the seed coat competes with the same nutrients supplied by the parent plant for the synthesis of storage reserves in the embryo and endosperm. For example, malonyl-CoA is a substrate for the synthesis of both flavonoids and FAs [18]. A regulatory mechanism is therefore required to coordinate the carbon partitioning for these metabolic pathways in the seed.

Several transcription factors that regulate seed coat development have been identified in Arabidopsis, including the *TRANSPARENT TESTA* (*TT*) genes, which encode MYB and bHLH transcriptional factors [15]. These MYB and bHLH proteins, together with TTG1 (WD40 family protein), can form ternary protein complexes named MBW [19], although the functions of the MBW complexes are not yet fully understood and additional transcription factors may interact with AC-rich MYB-binding sites [20]. The specific accumulation of PAs in the innermost cell layer of the seed coat involves at least four MBW complexes with partially overlapping functions [21]. Ectopic expression of MBW partners can activate not only the late biosynthetic genes, but also the entire flavonoid pathway [21, 22]. Previous comparative transcriptomic analysis in seed coats shows that genes in the phenylpropanoid and flavonoid biosynthesis pathways are downregulated in yellow seed coats compared to brown-seeded rapeseed [23]. Consistent with this, altered flavonoid metabolism in the seed coat affects the content and composition of storage compounds in the embryo. The *BnTT8* double mutants produce seeds with increased seed oil and protein content and altered FA composition [24], while yellow seed coat in *Brassica species* is also due to the loss of function of the *TT8* gene [25, 26]. Silencing of *BnTT1* resulted in a significant decrease in oleic acid (C18:1) and a notable increase in linoleic acid (C18:2) and linolenic acid (C18:3) in mature seeds [27]. These reported mutants are all accompanied by the changes in seed coat color and FA content, suggesting a negative correlation between seed coat flavonoid and FA accumulation.

In this work, we aimed to investigate the genetic mechanisms that may regulate the metabolic pathways for flavonoid and oil biosynthesis during seed development in *B. napus*. We analyzed a large set of rapeseed transcriptomes at 26 time points with a 2-day interval resolution during seed development. Combining the co-expression network analysis and the association studies of seed oil content (SOC) and seed coat content (SCC) in a natural variation population, we found that there was a wide range of co-expression between phenylpropane metabolism genes and oil content-related genes. A few phenylpropane metabolism genes were found to be inversely correlated with SOC. Our study provides new insights into the transcriptional regulation of carbon source competition between seed coat and embryo during *B. napus* seed development.

## Results

### The generation of seed transcriptomes of *B. napus*

Previously, we performed RNA-seq of 91 different rapeseed tissues from different developmental stages and constructed the BnTIR online server [28, 29], which has been integrated into BnIR [30]. In this database, 1.71 billion high-quality reads were generated from developing seeds (14–64 DAF with 2-day intervals) using the Illumina sequencing platform. These reads were then mapped to the ZS11 reference genome [31, 32] (Additional file 1: Fig. S1). On average, 96.6% of the reads were mapped (Additional file 2: Table S1), which were then used to calculate the normalized gene expression level as transcripts per million mapped reads (TPM). The sequencing data of the biological replicates were of high quality with Pearson correlation coefficients (*R*^2^) > 0.90, and seed development was clustered into several stages according to the distribution of high correlations (Additional file 1: Fig. S2, Fig. S3a; Additional file 2: Table S2). Thus, we took the average TPM value of three replicates as the expression level for each tissue.

### Seed transcriptomes are distinguished in five seed developmental stages

**Fig. 1:**
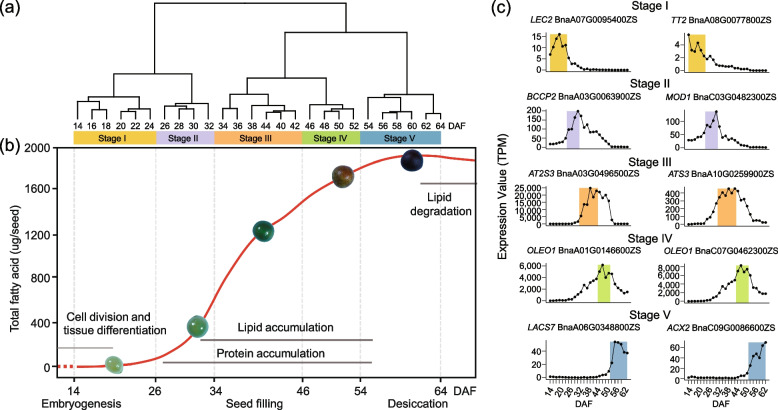
Transcriptome analysis of the 26 time points of *B. napus* seed development. **a** Cluster dendrogram showing five distinct development stages: embryogenesis, seed filling (rapid accumulation period and stable period), maturation, and desiccation during seed development. **b** Graphic representation of the FA content at five distinct seed development stages. A time course of major storage compound (e.g., seed storage lipid and protein) contents are shown in parallel. **c** Genes mainly expressed at the five different stages. The time points belong to the stages of embryogenesis, rapid seed filling, stable seed filling, maturation, and desiccation are shown in light yellow, light purple, deep yellow, green, and blue, respectively

With hierarchical clustering and principal component analysis (PCA), these high-density time-series transcriptomes could be generally divided into five clusters, with each cluster corresponding to a specific developmental stage, consistent with the previously reported timing of embryogenesis (stage I), seed filling (stages II and III, rapid accumulation period and stable period), preparatory desiccation phase (stage IV), and desiccation (stage V) during seed development (Fig. 1a, b; Additional file 1: Fig. S3b) [7, 33]. By quantifying the total FAs in developing seeds, we found that the total FAs started to accumulate around 20 DAF (stage I), followed by a period of rapid accumulation after 30 DAF (stage II), and reached a stable level before it started to decrease around 60 DAF (stage V) (Fig. 1b).

In order to further explore the characteristics of physiological changes during seed development, we analyzed the genes specifically expressed in seeds by Tau. A total of 5506 seed-specific expressed genes were identified, including 507 transcription factors (Additional file 1: Fig. S4; Additional file 2: Table S3). The genes specifically expressed at different stages were also screened according to the gene expression profile in seeds (Additional file 2: Table S4). For example, the genes from 14 to 24 DAF formed the first cluster, which represented the later stage of embryogenesis (stage I). Transcription factor *LEAFY COTYLEDON 2* (*LEC2*) that contains a B3 domain, a DNA-binding motif unique to plants and characteristic of several transcription factors, plays critical roles in both early and late embryo development [34]. *LEC2*, which is mainly expressed during seed development, is required for the maintenance of suspensor morphology, specification of cotyledon identity, progression through the maturation phase, and suppression of premature germination [35]. *TRANSPARENT TESTA 2* (*TT2; MYB123*) is expressed in embryos at an early developmental stage in *Arabidopsis* seeds and regulates embryonic FA biosynthesis by targeting *FUSCA3 (FUS3)* [36]. Several homologs of these two genes showed high expression after 14 DAF, but rapidly decreased after 26 DAF (Fig. 1c; Additional file 1: Fig. S5a), suggesting that *BnLEC2s* and *BnTT2s* might be important for embryonic morphologic development. Similarly, the genes from 26–32 DAF, 34–44 DAF, 46–52 DAF, and 54–64 DAF samples formed the second to fifth clusters, representing the stages of seed filling (rapid accumulation period, stage II), stable stage of seed filling (stage III), preparatory phase of seed desiccation (stage IV), and seed desiccation (stage V), respectively (Fig. 1c; Additional file 1: Fig. S5b-h). During these phases, the lipid biosynthesis genes (e.g., *Biotin carboxyl carrier protein* (*BCCP*), *MOSAIC DEATH 1* (*MOD1*)), seed storage protein genes (e.g., *2S albumins 3* (*2S3*), *Oleosin1* (*OLEO1*)), and phenylpropane metabolism pathway genes (e.g., *TRANSPARENT TESTA 12* (*TT12*), *BANYULS/ANTHOCYANIDIN REDUCTASE* (*BAN/ANR*)) were expressed (Additional file 1: Fig. S5; Additional file 2: Table S4). The temporal expression patterns of these genes in the five stages suggest their functions in FA biosynthesis and other metabolic processes during seed development and maturation.

### Co-expression network analysis highlights the gene modules of five stages

**Fig. 2:**
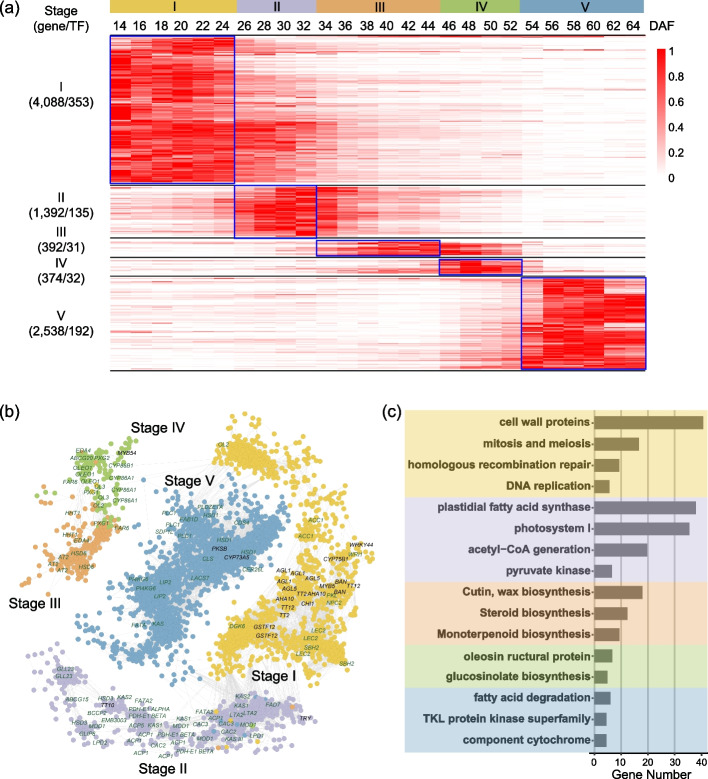
Co-expression network and function enrichment of five stages of seed development in *B. napus*. **a** Expression patterns of genes in five co-expression modules. For each gene, the TPM value normalized by the maximum value of all TPM values of the gene over all time points is shown. The number of genes and the transcription factors in each module are shown on the left. **b** Co-expression network of genes involved at the five stages of seed development in *B. napus*. Representative genes with known functions and TF genes involved in acyl-lipid metabolism are labeled. **c** MapMan function enrichment of genes at the five stages of seed development

The high temporal-resolution transcriptomes provided us with a good opportunity to identify genes and gene networks in different seed developmental stages that may provide information on the gene functions and genetic control of developmental phase transition. We then performed weighted gene co-expression network analysis (WGCNA) to identify highly interconnected modules of co-expressed genes in 26 seed developmental transcriptomes [37]. Modules were defined as clusters of highly interconnected genes and genes within the same cluster that had high correlation coefficients. The WGCNA analysis resulted in 17 distinct modules (labeled by different colors) composed of 14,964 genes (Additional file 2: Table S5) including 1265 transcription factors, 707 acyl-lipid-related genes (Additional file 2: Table S6), and 27 phenylalanine-related genes (Additional file 2: Table S7), in which each tree branch constitutes a module and each leaf in the branch is one gene (Additional file 1: Fig. S6a). The module eigengene was the first principal component of a given module and could be considered a representative of the module’s gene expression profile. The 17 module eigengenes for the 17 distinct modules were each correlated with distinct stage types due to eigengenes’ time-specific expression profiles. Notably, 5 out of the 17 co-expression modules comprised genes that were highly expressed in a single stage, and each of the five stages of developing seeds had a specific module (Fig. 2a, b; Additional file 1: Fig. S6b; Additional file 2: Table S5).

To further understand the functional characteristics of the five stages, we performed gene enrichment analysis for five stages and modules with MapMan (v3.6.0) [38]. In the five developmental stages, 84 significantly enriched MapMan terms were identified among all genes, showing highly stage-dependent patterns. Therefore, each of these five modules identifies (or correlates with) a specific stage cluster of genes (Fig. 2b, c; Additional file 1: Fig. S7; Additional file 2: Tables S8-S12). For example, the floral white module in stage I involved 4088 genes including 353 transcription factors (Fig. 2b, c; Additional file 2: Table S8), and the brown2 module in stage II identified 1392 genes including 135 transcription factors (Fig. 2b, c; Additional file 2: Table S9). Embryonic development-associated terms, “mitosis and meiosis,” “homologous recombination repair,” “cyclin activities,” “DNA replication,” and “cell wall organization,” were enriched in stage I (Fig. 2c; Additional file 2: Table S8), and “plastidial FA synthase,” “photosystem I,” “acetyl-CoA generation,” and “pyruvate kinase” were enriched in stage II (Fig. 2c; Additional file 2: Table S9).

### The phenylpropane metabolism pathway-related genes are associated with SOC in *B. napus*

**Fig. 3:**
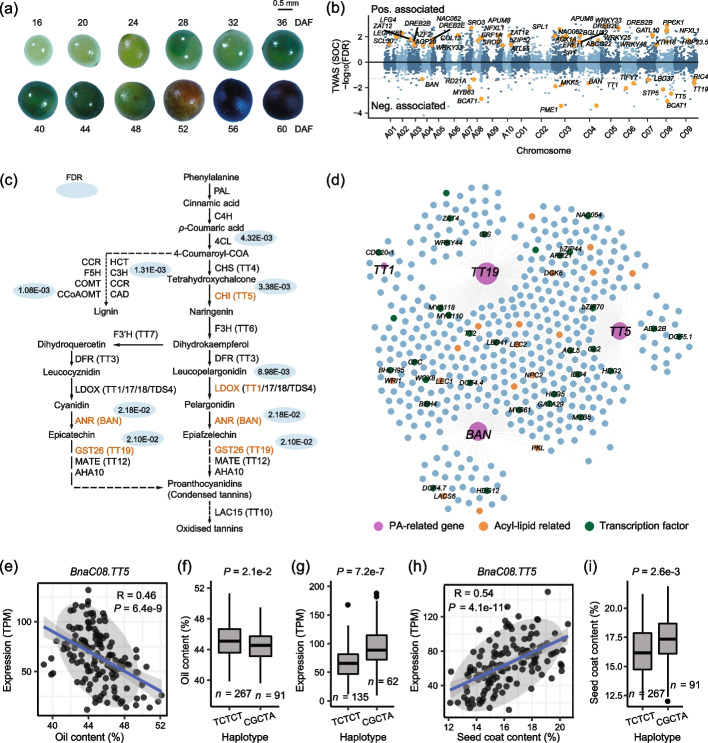
Procyanidin biosynthesis pathway was prominent during seed development. **a** Seed samples from 16 to 60 DAF (bar = 0.5 mm). **b** Manhattan plot of TWAS results (FDR < 0.05; [39]) for SOC. Genes with the expression positively or negatively correlated with SOC are plotted above or under the black bold line. Each point represents a single gene tested. The yellow points indicate overlapped genes between the co-expression network hub genes and TWAS-significant genes. **c** TWAS-significant genes (FDR < 0.05) involved in PAs and lignin biosynthesis pathway are marked in red, and the FDRs are shown in blue circles. **d** Co-expression network of *TT5*, *BAN*, *TT19*, and *TT1*. Circles in orange represent acyl-lipid genes, and circles in green represent transcription factor genes. Correlation between the expression (TPM) of *BnaC08.TT5* with oil content (**e**) and seed coat content (**h**). Box plots for oil content (**f**) and seed coat content (**i**) for the two haplotypes (*n* = 267 versus 91). **g** Box plots for the expressions for the two haplotypes (*n* = 135 versus 62). Center line, median; box limits, upper and lower quartiles; whiskers, 1.5 × the interquartile range; dots, outliers (*P* < 2e − 16, Student’s *t* test)

The high temporal-resolution transcriptomes allowed us to investigate the expression of different genes in the process of seed development. During seed development, many metabolites are accumulated or consumed and accompanied by changes in appearance, such as the color of the seed coat changed from light green to black due to the accumulation of oxidized PAs (Fig. 3a). We previously sequenced 583 seed transcriptomes of a natural *B. napus* population at two developmental stages and identified 692 genes significantly associated with SOC by transcriptome-wide association study (TWAS) [39–41]. We wonder whether the genes related to phenylpropane metabolism are associated with seed oil content. We used two methods to integrate the results from TWAS and co-expression networks (see the “Methods” section). In total, 12 hub genes were detected simultaneously by two methods (Fig. 3b; Additional file 2: Table S13-14).

As expected, several genes related to procyanidins, such as *TRANSPARENT TESTA 1* (*TT1)*, *TT5 (CHALCONE ISOMERASE (CHI))*, *TT19 (GLUTATHIONE S-TRANSFERASE PHI 12 (GSTF12))*, and *BANYULS* (*BAN)*, were significantly associated with SOC (Fig. 3b; Additional file 2: Table S14). TT5 and BAN are key enzymes in PA biosynthesis, while TT19 is a PA transporter (Fig. 3c). These three genes were co-expressed with hundreds of genes including acyl-lipid-related genes such as *LEAFY COTYLEDON 1* (*LEC1*, *NFYB9*), *LEC2*, *WRINKLED 1* (*WRI1*), *NON-SPECIFIC PHOSPHOLIPASE C2* (*NPC2*), *PICKLE* (*PKL*), *LACS6*, and *DIACYLGLYCEROL KINASE 6* (*DGK6*), as well as lipid degradation genes such as *LACS6*, *GDSL* (*AT1G71250*), and *lipase* (*AT1G18460*) (Fig. 3d; Additional file 2: Table S15-S16; Additional file 1: Fig. S8). Interestingly, we found that the expression of *TT5* (BnaC08G0351900ZS, *BnaC08.TT5*), *BAN* (BnaA03G0584100ZS, *BnaA03.BAN*), and *TT19* (BnaC09G0492500ZS, *BnaC09.TT19*) was negatively associated with SOC (Fig. 3e; Additional file 1: Fig. S9a-c) and highly expressed at the early stage of seed development (Additional file 1: Fig. S9d-i). In the natural population of *B. napus*, two major haplotypes, hap.TCTCT and hap.CGCTA, were identified in *BnaC08.TT5* (Additional file 1: Fig. S10a). In hap.CGCTA, the mutations of five bases were identified referring to hap.TCTCT, which caused five missense mutations of amino acids from T-G-N-E-Q to A-A-S-K-H (Additional file 1: Fig. S10a). The SOC of hap.TCTCT accessions was significantly higher than that of hap.CGCTA accessions (Fig. 3f), while the expression of *BnaC08.TT5* hap.TCTCT accessions was significantly lower than that of hap.CGCTA accessions (Fig. 3g). TT5 functions as a key enzyme involved in the synthesis of flavonoids, which may influence the deposition of these compounds in the seed coat [42]. We therefore analyzed the correlation between TT5 and SCC. As predicted, the expression of *BnaC08.TT5* was positively correlated with SCC (Fig. 3h), and the SCC of hap.TCTCT accessions was significantly lower than that of hap.CGCTA accessions (Fig. 3i). These results suggest that *BnaC08.TT5* is negatively associated with SOC and positively associated with SCC.

**Fig. 4:**
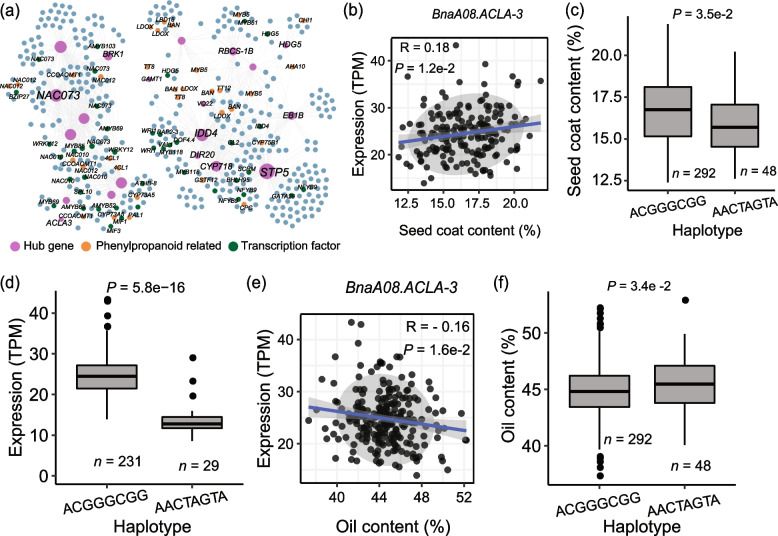
Co-expression analysis of phenylpropane metabolic genes. **a** Co-expression network of Hub genes in the phenylpropanoid genes network. Correlation between expression (TPM) of *BnaA08.ACLA-3* with seed coat content (**b**) and oil content (**e**). Box plots for seed coat content (**c**) and oil content (**f**) for the two haplotypes (*n* = 292 versus 48). **d** Box plots for the expressions for the two haplotypes (*n* = 231 versus 29). Center line, median; box limits, upper and lower quartiles; whiskers, 1.5 × the interquartile range; dots, outliers (*P* < 2e − 16, Student’s *t* test)

It has been shown that the content of phenylpropane metabolites in the seed coat, such as flavonoids and lignin, determines the color and SCC, which are closely correlated with oil content [43–46]. To further analyze the transcriptional regulation between phenylpropane metabolism and oil accumulation, the co-expression network was constructed using all phenylpropane metabolite synthesis genes. Among the top 100 hub genes, the expression of 36 genes was correlated with SCC in the natural population [47]. Among them, the expression of twenty genes was significantly correlated with SOC and SCC (Fig. 4a; Table 1). For example, *BnaA08.ACLA-3* (BnaA08G0294900ZS) was positively associated with SCC (Fig. 4b). *ACLA-3* encodes subunit A of the heteromeric enzyme ATP citrate lyase (ACL) that converts citrate to acetyl-CoA, which serves both as an immediate substrate for de novo lipogenesis (DNL) and an allosteric inhibitor of FAs oxidation [48]. There were two major haplotypes of *BnaA08.ACLA-3*, hap.CGGGCGG and hap.ACTAGTA, in the natural population (Fig. 4c). In hap.ACTAGTA, the mutations of seven bases were identified referring to hap.CGGGCGG, which caused seven missense mutations of amino acids from A-P–T-D-F-G-R to S-A-N–N-L-V-K (Additional file 1: Fig. S10b). The expression of hap.ACGGGCGG accessions was significantly higher than that of hap.AACTAGTA accessions (Fig. 4d). While *BnaA08.ACLA-3* was negatively associated with SCC (Fig. 4e), the SOC of hap.ACGGGCGG accessions was significantly lower than that of hap.AACTAGTA accessions (Fig. 4f). These results suggest that the expression of *BnaA08.ACLA-3* was correlated with SOC and SCC.

**Table 1:** Summary of candidate genes having a significant correlation with seed oil content and seed coat content in the co-expression network of phenylpropane-related genes

**Gene**	**Seed oil content**		**Seed coat content**		**Darmor gene**	***Arabidopsis* gene**	**Symbol**	**Transcription factor**
	**PCC**^a^	***P*-value**	**PCC**	***P*-value**				
BnaA02G0163400ZS	0.1858	0.0015	− 0.2459	0.0001	BnaA02g12810D	AT5G38430	RBCS-1B	–
BnaA02G0336300ZS	− 0.1786	0.0048	0.1377	0.0468	BnaA02g27910D	–	–	–
BnaA03G0370700ZS	− 0.2747	0	0.3424	0	BnaA03g36520D	AT3G22160	VQ22	–
BnaA05G0033300ZS	− 0.1768	0.0486	0.4166	0	BnaA05g34230D	AT2G42850	CYP718	–
BnaA05G0330700ZS	0.2963	0.0134	− 0.3255	0.0105	BnaA05g16630D	AT3G23090	–	–
BnaA08G0068400ZS	− 0.2676	0.0002	0.4331	0	BnaA08g29710D	AT1G34580	STP5	–
BnaA08G0294900ZS	− 0.1615	0.0132	0.1704	0.0175	BnaA08g26420D	AT1G09430	ACLA-3	–
BnaA09G0210400ZS	0.1702	0.0111	− 0.219	0.0025	BnaAnng07300D	AT5G46880	HDG5	HD-ZIP
BnaA09G0461900ZS	− 0.2985	0	0.3258	0	BnaA09g30660D	AT1G22410	–	–
BnaC01G0103400ZS	0.2117	0.0054	− 0.1911	0.0217	BnaC01g09920D	AT4G28500	NAC073	NAC
BnaC01G0446100ZS	− 0.1675	0.0043	0.3083	0	BnaC01g37520D	AT1G55210	DIR20	–
BnaC02G0432700ZS	− 0.1501	0.0097	0.1364	0.0304	–	AT2G02080	IDD4	C2H2
BnaC03G0110800ZS	− 0.1675	0.0115	0.1438	0.0449	BnaC03g11150D	AT5G60720	–	–
BnaC03G0553200ZS	− 0.1154	0.0472	0.1337	0.0353	BnaC03g74810D	AT5G62500	EB1B	–
BnaC04G0035900ZS	− 0.2249	0.0041	0.3451	0	BnaC04g52650D	AT2G42860	–	–
BnaC05G0194900ZS	− 0.3038	0	0.351	0	BnaC05g17710D	AT1G22410	–	–
BnaC08G0026700ZS	0.1598	0.0058	− 0.1663	0.0087	BnaCnng77910D	AT1G07120	–	–
BnaC08G0027400ZS	0.1466	0.0116	− 0.175	0.0057	BnaC08g02000D	AT1G07120	–	–
BnaC08G0169300ZS	− 0.2199	0.0002	0.3051	0	BnaC08g12180D	AT4G26420	GAMT1	–
BnaC08G0442600ZS	0.1663	0.0041	0.1395	0.0268	BnaC04g35160D	AT2G22640	BRK1	–

^a^ Pearson correlation coefficient

### The FA metabolism-related genes are associated with SCC in *B. napus*

**Fig. 5:**
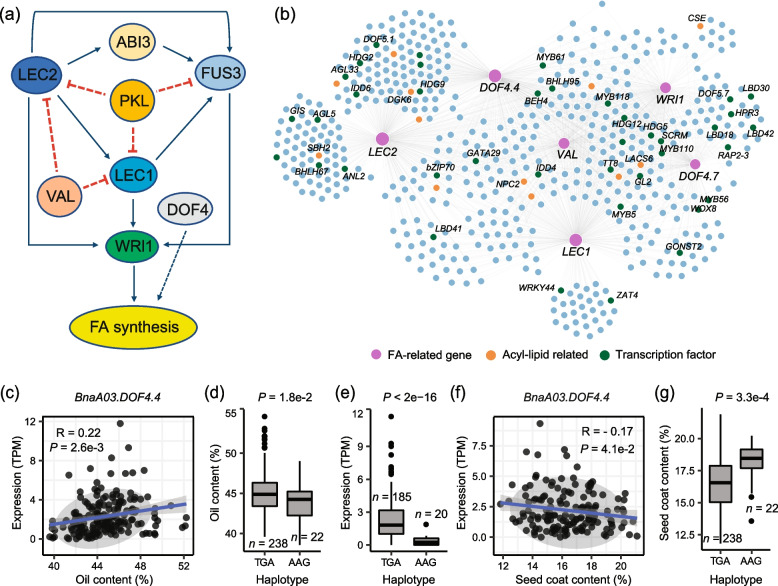
Co-expression network of key transcription factors of FA biosynthesis and haplotypes for the gene *DOF4.4* (BnaA03G0459300ZS). **a** Simplified regulatory network of FA biosynthesis in *Arabidopsis*. The blue line indicates a positive relationship. The red dashed line indicates a negative relationship. **b** Co-expression network of *LEC1*, *LEC2*, and *WRI1*. To avoid duplication, networks of different homolog genes are incorporated. Circles in orange represent acyl-lipid genes, and circles in green represent the transcription factor genes. Correlation between the expression (TPM) of *BnaA03.DOF4.4* with oil content (**c**) and seed coat content (**f**). Box plots for oil content (**d**) and seed coat content (**g**) for the two haplotypes (*n* = 238 versus 22). **e** Box plots for the expression for the two haplotypes (*n* = 185 versus 20). Center line, median; box limits, upper and lower quartiles; whiskers, 1.5 × the interquartile range; dots, outliers (*P* < 2e − 16, Student’s *t* test)

Four homologs of *LEC2*, three homologs of *LEC1*, and one homolog of *WRI1* were co-expressed with *TT5*, *TT19*, and *BAN* (Fig. 3d; Additional file 2: Table S15). Different homologs in *B. napus* may function in a coordinated way [49]. The co-expression correlation between *LEC1* and *LEC2* homologs was strong (Additional file 1: Fig. S11a), as 96.88–99.74%, and 90.38–99.92% of the genes were present in at least two co-expressed networks of *LEC1* and *LEC2* homologs, respectively (Additional file 1: Fig. S11b, c). LEC1, LEC2, and WRI1, together with FUS3, ABSCISIC ACIDINSENTIVE3 (ABI3), and other transcription factors, constitute the core regulatory network of FA biosynthesis [34, 50–56] (Fig. 5a). Subsequently, we constructed the gene regulatory network of these two genes with an incorporated connection (Fig. 5b). In addition, the gene regulatory network connects *LEC1*, *LEC2*, and *WRI1* with their co-expressed genes including a number of acyl-lipid related genes such as *VIVIPAROUS1/ABI3-LIKE1* (*VAL1*), *DNA-BINDING WITH ONE FINGER 4* (*DOF4*), *PKL*, *NPC2*, and *DGK6* (Fig. 5b).

In the co-expression network of FA biosynthesis genes, 84 genes were significantly correlated with SOC and SCC (Additional file 2: Table S17). As expected, *B. napus LEC1*, *LEC2*, and *WRI1* were positively associated with SOC (Fig. 5c; Additional file 1: Fig. S12a-c). The expression peak of these genes was different (*LEC2*, 14 to 32 DAF; *LEC1*, 14 to 40 DAF; *WRI1*, 14 to 56 DAF) (Additional file 1: Fig. S12d-i), whereas other acyl-lipid-related genes including *NPC2*, *LACS6*, and *DGK6* were mainly expressed during stages I–II (Additional file 1: Fig. S13). All these genes were almost no longer expressed after 56 DAF, consistent with the FA accumulation at stage V (Fig. 1b). Besides the acyl-lipid-related genes, transcription factors of the MYB, WRKY, bHLH, and ZAT families also appeared in the co-expression networks. Among them, MYB118 has been reported to repress the expression of maturation-related genes in the endosperm of *Arabidopsis* seeds [57, 58]. ZAT4 is a typical C2H2-type transcription factor that plays an important role in stress tolerance [59, 60]. *BnaA04G.ZAT4* (BnaA04G0285000ZS) showed a negative correlation with SOC and a positive correlation with SCC; the SOC of hap.CA accessions were significantly higher than that of hap.TG accessions, while the expression and the SCC of hap.CA accessions were significantly lower than that of hap.TG accessions (Additional file 1: Fig. S14). Plant-specific DNA-binding with one finger (DOF)-type transcription factors regulate various biological processes [61–63]. AtDOF4.4 plays major roles in shoot branching and seed/silique development [63]. We found that *BnaA03.DOF4.4* (BnaA03G0459300ZS) was positively associated with SOC. *BnaA03.DOF4.4* contained two haplotypes; in hap.AAG, the mutations of three bases were identified referring to hap.TGA, which caused three missense mutations of amino acids from V-S-M to D-N-V (Additional file 1: Fig. S15). The SOC of hap.TGA accessions was significantly higher than that of hap.AAG accessions (Fig. 5d), while the expression of hap.AAG accessions was significantly lower than that of hap.TGA accessions (Fig. 5e). Interestingly, *BnaA03.DOF4.4* was negatively associated with SCC (Fig. 5f), and the SCC of hap.TGA accessions was significantly lower than that of hap.AAG accessions (Fig. 5g). Another DOF gene *BnaC01.DOF4.7* also showed similar correlations with SOC and SCC as DOF4.4 (Additional file 1: Fig. S16).

**Fig. 6:**
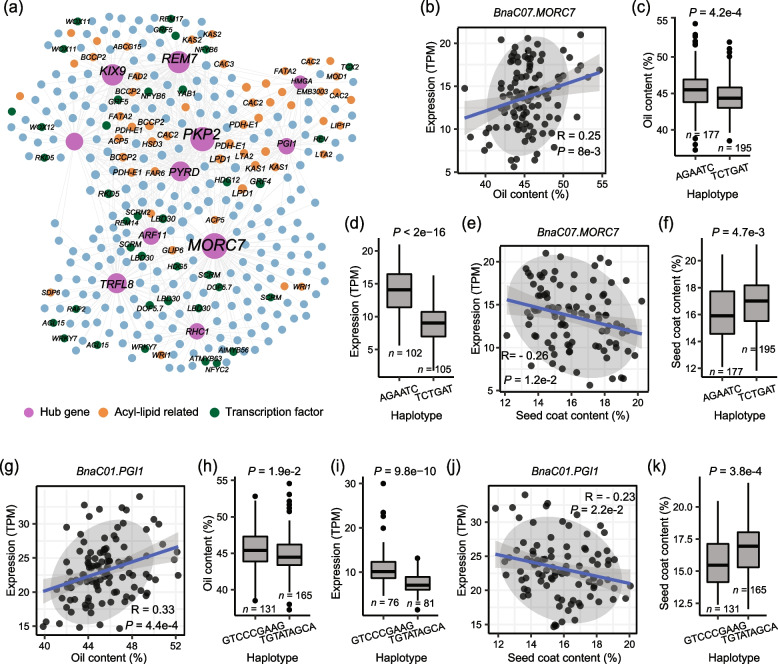
Co-expression analysis of FA biosynthesis hub genes correlated with both SOC and SCC. **a** Co-expression network of hub genes in the acyl-lipid genes network. Correlation between the expression (TPM) of *BnaC07.MORC7* with oil content (**b**) and seed coat content (**e**). Box plots for oil content (**c**) and seed coat content (**f**) for the two haplotypes (*n* = 177 versus 195). **d** Box plots for the expressions for the two haplotypes (*n* = 102 versus 105). Correlation between the expression (TPM) of *BnaC01.PGI1* with oil content (**g**) and seed coat content (**j**). Box plots for oil content (**h**) and seed coat content (**k**) for the two haplotypes (*n* = 131 versus 165). **i** Box plots for the expressions for the two haplotypes (*n* = 76 versus 81). Center line, median; box limits, upper and lower quartiles; whiskers, 1.5 × the interquartile range; dots, outliers (*P* < 2e − 16, Student’s *t* test)

We also constructed the co-expression network using FA biosynthesis genes [64]. In the top 100 hub genes, the expression of 17 genes was correlated with SCC in the natural population. Among them, the expression of twelve genes was significantly correlated with both SOC and SCC, and the co-expression networks of these genes were further mapped (Fig. 4a; Additional file 2: Table S18). Many FA synthesis genes were co-expressed with these hub genes, including *BCCP2*, *FAD2*, *FATA2*, *KAS1*, and *WRI1* (Fig. 6a). Correlation analysis of genes with oil content showed that *BnaC07.MORC7* was positively correlated with SOC (Fig. 6b). In *Arabidopsis*, MORC family ATPases catalyze changes in chromatin structure, inducing gene silencing via RNA-directed DNA methylation, and MORC7 can suppress genes in a methylation-independent manner [65, 66]. At the population level, two major haplotypes, hap.AGAATC and hap.TCTGAT, were identified in *BnaC07.MORC7* (Fig. 6c). In hap.TCTGAT, the mutations of six bases were identified referring to hap.AGAATC, causing six missense mutations of amino acids from I-S-M–K-S-P to F-T-L-E-R-L (Additional file 1: Fig. S17a). The expression of hap.AGAATC accessions was significantly higher than that of hap.TCTGAT accessions (Fig. 6d), while the SOC of hap.AGAATC accessions was significantly higher than that of hap.TCTGAT accessions (Fig. 6c). Meanwhile, *BnaC07.MORC7* was negatively associated with SCC (Fig. 6e). The SCC of hap.AGAATC accessions was significantly lower than that of hap.TCTGAT accessions (Fig. 6f). Similarly, *BnaC01.PGI1* (BnaC01G0181100ZS), a paralog of *Arabidopsis PGI1*, shows correlations with SOC and SCC, containing two major haplotypes in natural population including hap.GTCCCGAAG and hap.TGTATAGCA (Fig. 6g–k). In a natural population, in hap.TGTATAGCA, the mutations of nine bases were identified referring to hap.GTCCCGAAG, which caused four missense mutations of amino acids from E-V-M-T to K-A-R-I (Additional file 1: Fig. S17b). These results suggest that the expression of some genes related to FA biosynthesis also affects the SCC.

### Prediction of candidate transcription factors which impact SOC

**Fig. 7:**
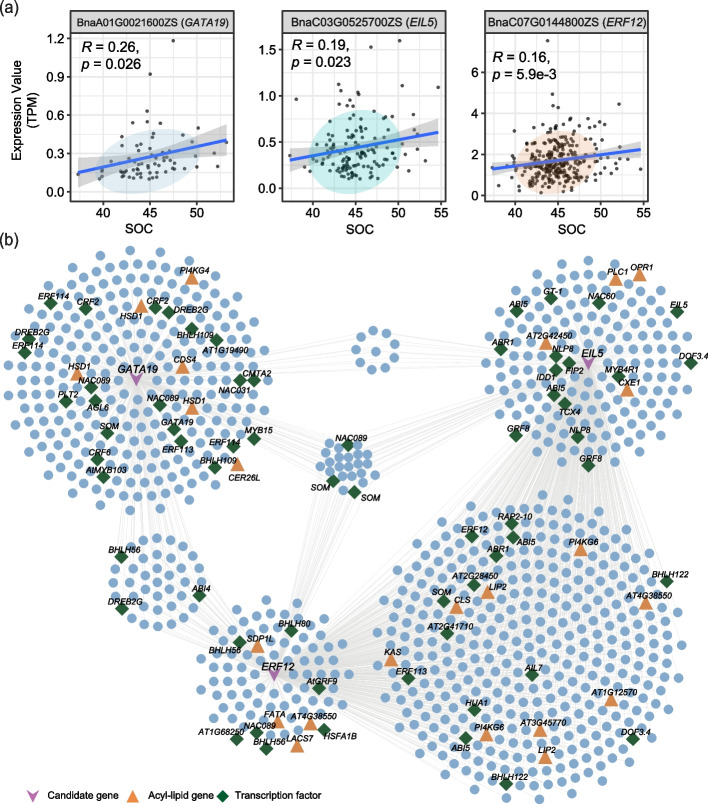
Novel candidate transcription factors that may regulate SOC. **a** Correlation between the expression of seed-specific transcription factors (*GATA19*, *EIL5*, and *ERF12*) and SOC. **b** Genes regulated by network hubs (*GATA19*, *EIL5*, and *ERF12*). Three candidate TF genes are drawn as V’s in purple. Triangles in orange represent acyl-lipid genes, and the diamonds in green represent transcription factor genes

To investigate whether the 507 seed-specific expressed transcription factors modulate FA biosynthesis, we analyzed seed-specific TF genes, TWAS-significant SOC genes, and co-expression network hub genes. The results showed that 309 TF genes were among TWAS-significant genes or co-expression network hub genes (Additional file 2: Table S19), and 51 of them were significantly correlated with SOC (Fig. 7a; Additional file 1: Fig. S18a-c; Additional file 2: Table S20). Among these overlapping seed-specific transcription factors, the top three were *EIL5*, *ERF12*, and *GATA19*, which were predicted to interact with 572, 519, and 341 genes, respectively (Fig. 7b; Additional file 2: Table S21). These three TFs were all highly expressed in stage V (Additional file 1: Fig. S18a-c). As shown in Fig. 7b, *EIL5*, *ERF12*, and *GATA19* and their co-expressed genes formed a closely related community. There were 968 genes including 28 acyl-lipid-related genes (*LACS7*, *SDP1L*, *LIP2*, *KAS*, *FATA*, *HSD1*, *OPR1*, *PI4KG4*, and *PLC1*) that were predicted to be simultaneously regulated by *EIL5*, *ERF12*, and *GATA19* (Additional file 2: Tables S21, 22). A total of 23 genes were simultaneously regulated by *EIL5*, *ERF12*, and *GATA19*, and the promoter of 15 genes contained transcription factor-binding sites (TFBS) of EIL, ERF, and GATA (Fig. 7b; Additional file 2: Table S23). Further studies on these transcription factors are required to elucidate their functions in the regulation of SOC.

## Discussion

Seed maturation is characterized by the accumulation of carbon reserves, including carbohydrates, protein, and oil. In *B. napus*, about 2.2% of genes in the genome are lipid metabolism genes, most of which are highly conserved among different species [67], which is twice as much as in soybean and oil palm [68, 69]. However, the genetic regulation of these genes in *B. napus* remains unclear, and even less is known about how oil biosynthesis is coordinated with other concurrent metabolic pathways in different compartments. Here, we revealed a high temporal-resolution transcriptome landscape spanning the major periods of seed development in *B. napus* by sampling at 26 time points from 14 to 64 DAF with 2-day intervals. This large collection of gene expression information provides a rich resource for understanding the genetic control of seed development and for designing effective strategies to increase the yield of storage compounds.

To improve oil yield and quality in rapeseed, yellow seed coat color is considered a desirable trait. Our analysis therefore focuses on the gene networks regulating the biosynthesis of oil in the embryo and specialized metabolites in the seed coat. The pathways for the biosynthesis of storage lipids and seed coat flavonoids are well known, and much knowledge has also been obtained for their genetic regulation [15, 70]. Our comprehensive seed transcriptomes allowed us to decipher the genetic mechanisms that regulate these key metabolic events in a time-dependent manner. Based on the co-expression network analysis of acyl-lipid-related genes across five stages during seed development, 1265 seed-specific transcription factors were identified in 17 modules. In the core module corresponding to the five stages, 135 transcription factors were specifically expressed in stage II, and their functions were enriched in “plastidial FA synthase,” “photosystem I,” “acetyl-CoA generation,” and “pyruvate kinase.” In addition to these transcription factors, several new transcription factors were predicated, such as *EIL5*, *ERF12*, and *GATA19*. In *Arabidopsis*, ERF12 is involved in floral organ development [71], and GATA transcription factors regulate shoot apical meristem and flower development in *Arabidopsis* [72, 73]. The expression level of these three transcription factors was highest at the late stage of seed development (Additional file 1: Fig. S18f-h). During the *B. napus* seed development, these three transcription factors and their interacting genes formed a closely related community, including 18 acyl-lipid-related genes (Fig. 7). These results suggest that these transcription factors might be involved in the FA biosynthesis; it also indicates that genes specifically expressed at stage V are also important for seed oil accumulation.

At the later stage of seed development, the seed lipid content of *B. napus* can be reduced by 10–14% [74]. This conclusion is supported by our oil content analysis (Fig. 1b), and genes involved in β-oxidation including *LACS7*, *ACX3*, and *ACX2* were enriched at stage IV (46–52 DAF) of seed development (Fig. 2c; Additional file 2: Table S4). TAG and FAs are catabolized by β-oxidation within the peroxisome to acetyl-CoA [75] and then subsequently converted to succinate via the glyoxylate cycle [76], providing germinating seeds with both carbon skeletons and energy before the seedlings develop the photosynthesis capacity. TAG degradation during *Arabidopsis* seed desiccation is also thought to be used to support continued metabolism after the carbon input from the maternal tissue has ceased. Indeed, during *Arabidopsis* embryo maturation, the trophic link between the seed and the maternal parent is lost before metabolic activity ceases [77]. However, the synthesis of storage proteins, the abundant proteins of late embryogenesis, and other metabolic processes associated with seed maturation continue until desiccation [77, 78]. Thus, the metabolism in late embryogenesis resembles that of carbon-deficient tissues in which FAs turnover can be activated [79]. In the embryonic development of *B. napus*, carbon from FA catabolism is explicitly utilized in the same tissue [74]. Therefore, we propose that during seed desiccation, lipid degradation may reserve intermediates for seed germination and other metabolic processes, while the carbon source competition between phenylpropane metabolism and oil accumulation should occur at the early stage of seed development. We also noticed that many of the candidate genes predicted by the co-expression network to be associated with SOC and SCC were not very highly correlated with both traits. This might be due to the structure of this population, which contains spring, semi-winter, and winter ecotypes [39]. On the other hand, SOC is a complex quantitative genetic trait controlled by multiple minor effect genes, and many factors could affect the SOC [80]. The contribution of each oil-related gene to SOC is small, which requires pyramiding of multiple genes to breed *B. napus* with high SOC.

The seed coat color is determined by the deposition of the phenylpropane compounds cyanidin and procyanidins (PAs) in their endodermal cells. As the seed coat cells die, PAs are released from the endothelial cells and immersed into the seed coat, giving the seed coat its deep color [15, 81]. PAs and other flavonoids are synthesized through the phenylpropanoid pathway, where phenylalanine is converted to 4-coumaroyl-CoA, which enters the flavonoid biosynthetic pathway to form PAs tannins and lignin [82, 83] (Fig. 3c). Phenylpropanoid homeostasis among different branches of phenylpropanoid metabolism, achieved through the regulation of metabolic flux redirection (MFR), shows extraordinary complexity and a high degree of plasticity during successive developmental stages and in response to environmental stimuli and changes [84]. For example, the balance between anthocyanin and lignin biosynthesis is regulated by the MdMYB16/MdMYB1-miR7125-MdCCR model integrating light signaling in apple [85]. In *Arabidopsis*, the flavonoid pathway has been well characterized at the molecular level, mainly using *transparent testa* (*tt*) mutants that affect flavonoid accumulation and seed coat color [86–89]. In *B. napus*, *TT1* and *TT8* have also been proven to affect flavonoid accumulation and seed coat color [24, 27].

**Fig. 8:**
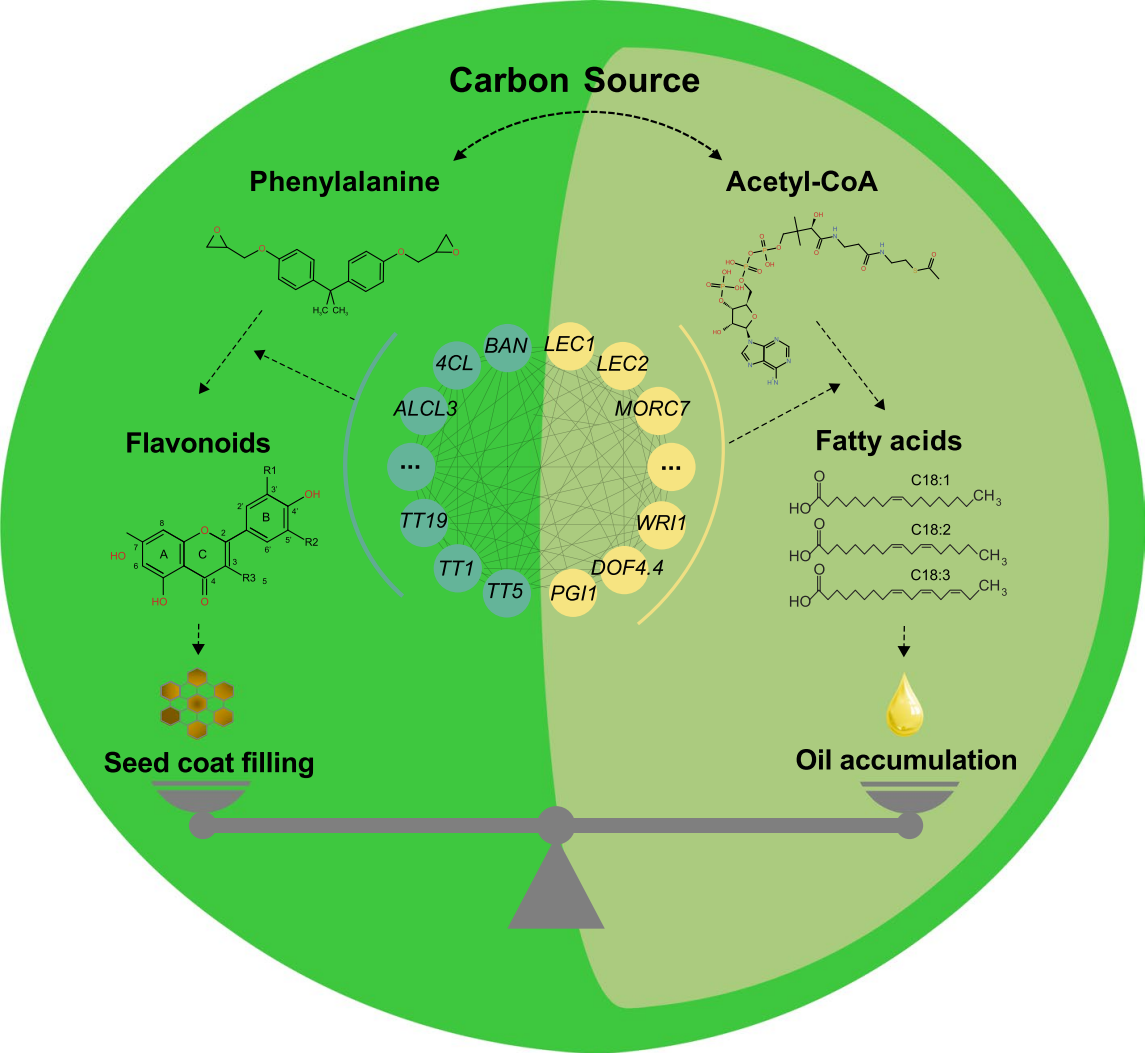
The model of carbon source competition between seed coat and embryo in developing seed of *B. napus*. During seed development, a part of the carbon source is allocated to the seed coat, and phenylalanine is metabolized into 4-coumarate-CoA to provide one of the substrates (another is malonyl-CoA) for the synthesis of flavonoid compounds and lignin monomers. At the later stage of seed development, oxidized procyanidins and anthocyanins act as important fillers in the endodermis of the seed coat, determining the seed coat color and thickness. Another part of the carbon source is converted to pyruvate by glycolysis to produce acetyl-CoA, which provides the direct substrate malonyl-CoA for the synthesis of FAs and further transformed into stored oil bodies after the synthesis of triacylglycerol. These two processes compete for initial sugar allocation and malonyl-CoA allocation, while the phenylpropane metabolic pathway and FA synthesis pathway, as two important processes determining seed coat development and oil accumulation, are controlled by a strict and complex regulatory mechanism operated by a series of transcription factors

Previous studies reveal that the seed coat of yellow-seeded *B. napus* is thinner than the black-seeded type, which contains higher oil and protein content [4]. We hypothesized that the higher oil content in yellow seeds than in brown/dark seeds may have resulted in the favorable carbon partitioning towards the embryo compared to the seed coat, since the synthesis of FAs and pigmentary flavonoid compounds uses the common source of carbon from the parent plant (Fig. 8). Sucrose translocated from photosynthetic tissues is the major carbon source in the developing seed. Hydrolysis of sucrose and subsequent glycolysis produce acetyl-CoA, which can be carboxylated to malonyl-CoA, both of which are substrates for FA biosynthesis, while malonyl-CoA is also used for flavonoid synthesis [18]. These clearly competing metabolic pathways must be tightly regulated to coordinate optimal seed storage reserves and proper seed coat development for protection. Our transcriptome and gene network analyses provide important insights. The dynamic transcriptomes during rapeseed development clearly demonstrated distinct stage-specific gene expression patterns corresponding to the major phases of late embryogenesis and the transition to seed maturation and accumulation of carbon reserves (Fig. 2). As both embryo and seed coat undergo rapid expansion and accumulation of storage compounds during these stages, we found extensive co-expression of key transcription factors and enzymes involved in oil (e.g., LEC2, LEC1, FUS3, and WRI1) and PA biosynthesis (e.g., TT2, TT2, TT19, and BAN). Importantly, we found that many genes involved in phenylpropane metabolism were significantly associated with SOC in the TWAS (Fig. 3c), and the co-expression network of these genes also included key genes involved in oil synthesis (Fig. 3d). PA biosynthesis-related genes such as *TT1*, *TT5 (CHI)*, *TT19 (GSTF12)*, and *BAN* were positively correlated with SCC but were negatively correlated with SOC (Additional file 1: Fig. S9a-c; Additional file 2: Table S17). This suggests that the seed coat formation together with flavonoid accumulation competes with FA biosynthesis using the fixed carbon source in the seed, thereby affecting the oil content. Our results are consistent with various evidence showing that the content of phenylpropane metabolites including flavonoids, lignin, and fiber in the seed coat can determine the seed coat color and SCC, which is often accompanied by the change of SOC [43–46, 90], which supports the above views.

## Conclusions

In summary, we have provided high temporal-resolution transcriptomes during *B. napus* seed development. The 26 time-course transcriptomes are clearly clustered into five distinct groups from stage I to stage V. Co-expression network analysis and correlation analysis revealed the carbon source competition between the seed coat and embryo, and these two processes were under fine regulation by a number of transcription factors during seed development. The co-expression genes of the phenylpropanoid and FA pathways, especially the transcription factor-target genes revealed here, provide valuable tools for optimizing carbon partitioning to achieve high yields of seed storage products through biotechnology.

## Methods

### Plant materials and sampling

The RNA-seq data were generated in our previous study [28]. All transcriptomes could be obtained from the BnIR database (https://yanglab.hzau.edu.cn).

### RNA isolation and transcriptome sequencing

Total RNA was extracted using TRIzol Reagent (Invitrogen Life Technologies, USA) according to the manufacturer’s instructions. A total amount of 1.5 μg RNA per sample was used to generate RNA-seq libraries by the TruSeq RNA Sample Preparation Kit (Illumina, San Diego, CA, USA). To select cDNA fragments of the preferred 200 bp in length, the library fragments were purified by the AMPure XP system (Beckman Coulter, Beverly, CA, USA). DNA fragments with ligated adaptor molecules on both ends were selectively enriched using Illumina PCR Primer Cocktail in a 15-cycle PCR reaction. Products were then purified (AMPure XP system) and quantified by the Agilent high-sensitivity DNA assay on a Bioanalyzer 2100 system (Agilent). Paired-end (PE) sequencing was performed on these libraries using next-generation sequencing (NGS) technology on the NovaSeq 6000 platform (Illumina) in PE150 mode by Shanghai Personal Biotechnology Co., Ltd.

### Read mapping and expression profiling

The quality of the RNA sequencing reads was examined by FastQC (v0.11.9) (https://www.bioinformatics.babraham.ac.uk/projects/fastqc/). Barcode adaptors and low-quality reads (read quality < 80 for paired-end reads) were removed by Trimmomatic (v0.38) [91]. Then, the filtered reads were aligned to the *B. napus* reference genome [31] and annotated by three different pipelines which were described below.

For the first pipeline, the filtered reads from each sample were mapped to the reference genome using Hisat2-2.1.0 [92] with default parameters. Bam files containing aligned reads were inputted into StringTie (v1.3.3b) [93] to measure the expression level of genes. For the second pipeline, the STAR (v2.7.5a) [94] software was used to map the reads to the reference genome. The junctions detected in the first pass were collected and used them as “annotated” junctions for the 2nd pass mapping. Reads or fragments were counted from BAM files using the FeatureCounts (v1.6.4) [95]. For the third pipeline, we used the Salmon (v1.3.0) [96] to build the index of the reference genome with default parameters. The TPM measure for each sample was used Salmon with the parameter “validateMapping” for preserving all the genes. The Pearson correlation coefficient between biological replicates was calculated by the R software using gene expression values from the first pipeline.

### PCA and hierarchical clustering

To facilitate the graphical interpretation of relatedness among 26 different time points of seed, we reduced the dimensional expression data to two dimensions by PCA using the “prcomp” function in the R software with default settings. Hierarchical clustering was performed by the pvclust package (v2.2.0) with a default setting using Pearson’s correlation distance [97]. The transformed and normalized gene expression values with log_2_(TPM + 1) were used for the analysis of PCA and hierarchical clustering.

### Identification of seed-specific gene expression

To identify genes that are preferentially expressed in seed tissues, RNA-seq data from 91 different tissue samples that include 26 seed samples harvested at different time points and 65 non-seed samples were used to calculate the gene expression level. Genes were categorized according to their expression level patterns and tau scores. Tau is one of the frequently used algorithms for estimating the *τ* value for screening tissue-specifically expressed genes [98, 99]. Genes expressed ≤ 1.0 TPM in all tissues were excluded from subsequent analysis. Then, expression levels were transformed based on log [2], and the tau score was calculated for each gene using the TBtools [100]. The values of tau vary from 0 to 1, and the genes having *τ* > 0.9 were classified as tissue-specific expression [98, 99].

### Identification of co-expression modules using WGCNA

We filtered genes by mean expression and variance before performing WGCNA. The details are as follows: (i) remove genes having a lower expression and retain the 50,000 most expressed genes and (ii) remove genes which expression is too similar across samples and retain the top 15,000 genes with a high coefficient of variance (https://horvath.genetics.ucla.edu/html/CoexpressionNetwork/Rpackages/WGCNA/faq.html) [101]. The WGCNA R package [102] was used with the filtered genes to define modules and gene connections. A matrix of pairwise Spearman correlation coefficient between all pairs of genes was generated and transformed into an adjacency matrix (a matrix of connection strengths) using the formula: connection strength (adjacency value) =|(1 + correlation)/2|^*β*^. Here, *β* represents the soft threshold for the correlation matrix. A *β* value of 12 was determined based on the scale-free topology criterion [103]. The resulting adjacency matrix was converted to a topological overlap (TO) matrix via the TOM similarity algorithm, and the genes were hierarchically clustered based on TO similarity. The dynamic tree-cutting algorithm was used to cut the hierarchal clustering dendrogram, and modules were defined after combining branches to reach a stable number of clusters. The Gephi v0.9 (http://gephi.org) software “MultiGravity ForceAtlas 2” layout was used to visualize the gene interactions and the network among the five seed development-related modules.

### Functional enrichment analysis of co-expression modules

Functional category enrichment for each co-expression module was evaluated by MapMan (v3.6.0RC1) functional annotation [38]. Before conducting the MapMan annotation, we used the protein sequences from ZS11 as a representative protein and ran the Mercator (http://mapman.gabipd.org/) with default settings. Fisher’s exact test was used to examine whether the functional categories were overrepresented for a given module. The resulting *P* values were adjusted to *Q* values by the Benjamini–Hochberg correction and a false discovery rate of 5% was applied.

### Acyl-lipid-related co-expression network

The co-expression network of acyl-lipid was built as the previous report [37]. The genes in co-expression modules were assembled in a “candidate-gene set.” A total of 885 *Arabidopsis* gene protein sequences involved in acyl-lipid metabolism (http://aralip.plantbiology.msu.edu/pathways/pathways) were used to search for their orthologs in *B. napus* through BLASTP 2.6.0 + program with a cutoff *e*-value < 10^−5^. The 3044 *B. napus* acyl-lipid genes were defined as a “guide-gene set,” which were used to build the co-expression network. The connections between guide genes and candidate genes were built by using the method previously reported [104]. All connections between guide genes and their linked partners (P1) and connections between P1 were computed. The gene was retained only if the absolute value of Pearson’s correlation coefficient (*R*) between genes was larger than 0.8. All transcription factors and the genes with the top 10% highest connectivity in each module were defined as hub genes [37]. The Cytoscape v3.6 software “yFiles Organic Layout” was used to visualize the acyl-lipid co-expression network when Pearson’s correlation coefficient between genes was larger than 0.9 [105].

### Integrate the results from TWAS and co-expression network

We used the “Overlap” and “cageminer” methods to integrate the results from TWAS and co-expression networks. “Overlap” method—(i) the 692 SOC-associated genes were identified by TWAS [39], and their orthologs in ZS11 were identified using the “Gene index” module in BnTIR [28]; (ii) the transcription factors and genes with the top 10% highest connectivity in each module were defined as co-expression hub genes [37]; and (iii) identified genes that were enriched in both co-expression hub genes and TWAS-significant genes. Furthermore, we also use the “cageminer” R package [106] to integrate the results of TWAS and co-expression network: (i) using SOC-associated genes identified by TWAS as putative candidate genes, (ii) using acyl-lipid (Additional file 2: Table S24) and phenylalanine (Additional file 2: Table S25) genes as guide genes; and (iii) The function “mine_step2()” in the “cageminer” R package was used to select candidates in the co-expression modules enriched in guide genes. In total, 59 genes were identified, and 12 genes were detected simultaneously by two methods.

### Quantitative RT-PCR analysis

Verification of differential gene expression was performed using RNA samples for sequencing. cDNA was synthesized using the Transcript RT Kit (TransGen Biotech). qPCR was carried out using the TransStart Top Green qPCR SuperMix Kit (TransGen Biotech) on a CFX384 Real-Time System (Bio-Rad). Relative quantification was performed using the comparative cycle threshold method, and the relative amount of PCR product that was amplified using the designed primer sets (Additional file 2: Table S26) was normalized to the reference gene *BnACT7*.

### Seed FA analysis

FAs of developing seeds were analyzed by GC-FID as previously described [107]. Briefly, a known number of seeds from each sample were dried and weighed, after that, FAs were methylated and extracted with methanol containing 5% (v/v) H_2_SO4 and 0.01% butylated hydroxyl toluene (BHT). FAs were quantified using a gas chromatograph and identified according to their retention times, and the peak area was used to quantify FA composition by comparing with the internal standard heptadecanoic acid (17:0). FA composition and total FAs were calculated as µg per seed.

### Supplementary Information

**Additional file 1: Fig. S1. **RNA-seq data analysis process and the core module of database.** Fig. S2. **Correlation between biological replications, four materials were randomly selected from the seed (Average R=0.97). **Fig. S3. **The correlation and PCA analysis across the transcriptomes of the 26 time points of seeds.** Fig. S4. **Expression patterns of seed-specific genes in all seed and non-seed samples.** Fig. S5. **Expression profile of marker genes in different stages.** Fig. S6. **WGCNA co-expression networks.** Fig. S7. **Enrichment analysis for genes in five core modules.** Fig. S8. **The co-expression network of *TT5* (a), *BAN* (b), *TT19* (c).** Fig. S9. **Correlation between SOC and expression of *TT5*, *BAN* and *TT19*.** Fig. S10. **Variation in the protein sequence of different haplotypes of *BnaC08.TT5* (a) and *BnaA08.ACLA-3* (b).** Fig. S11. **Correlations between the paralogues of *LEC2* and *LEC1*.** Fig. S12. **Correlation between SOC and expression of *LEC2*, *LEC1* and *WRI1*.** Fig. S13. **qPCR validation of FAs biosynthesis related genes.** Fig. S14. **Haplotypes for the gene *ZAT4* (*BnaA04G0285000ZS*).** Fig. S15. **Variation in the protein sequence of different haplotypes of *BnaA03.DOF4.4*.** Fig. S16. **Haplotypes for the gene *DOF4.7* (*BnaC01G0012600ZS*).** Fig. S17. **Variation in the protein sequence of different haplotypes of *BnaC07.MORC7* (a) and *BnaC01.PGI1* (b).** Fig. S18. **Expression profile of candidate genes.

**Additional file 2: Table S1. **Summary of RNA sequencing.** Table S2. **Correlation between biological replicates and different time points.** Table S3. **Summary of seed-specific genes.** Table S4. **Summary of marker genes in each core module.** Table S5. **Summary of genes in each co-expression network module.** Table S6. **Summary of acyl-lipid genes in each core module.** Table S7. **Summary of phenylalanine genes in each core module.** Table S8. **Summary of MapMan enrichment results of floralwhite (stage I) module.** Table S9. **Summary of MapMan enrichment results of brown2 (stage II) module.** Table S10.** Summary of MapMan enrichment results of palevioletred2 (stage III) module.** Table S11.** Summary of MapMan enrichment results of darkorange2 (stage IV) module.** Table S12.** Summary of MapMan enrichment results of midnightblue (stage V) module.** Table S13.** Summary of hub genes in each co-expression network module.** Table S14. **Summary of genes integrated from co-expression network and TWAS-significant genes.** Table S15.** Summary of acyl-lipid genes in *TT5*, *BAN* and *TT19* co-expression network.** Table S16.** Summary of acyl-lipid genes in TT5, BAN, TT19 and TT1 co-expression network.** Table S17.** Summary of candidate genes that expression significant correlation with seed oil content and seed coat content in co-expression network of FAs biosynthesis genes.** Table S18. **Summary of candidate genes that expression significant correlation with oil content and seed coat content in co-expression network of acyl-related genes.** Table S19.** Seed-specific transcription factors overlapped with TWAS-significant genes or co-expression network hub genes.** Table S20. **Seed-specific transcription factors that significantly correlated with SOC.** Table S21.** Summary of genes in *EIL5, ERF12 and GATA19* co-expression network.** Table S22.** Summary of acyl-lipid genes in *EIL5, ERF12 and GATA19* co-expression network.** Table S23. **The binding site information in the promoter of genes that are coregulated by *EIL5, ERF12 and GATA19*.** Table S24.** Summary of acyl-lipid genes in ZS11.** Table S25.** Summary of phenylalanine genes in ZS11.** Table S26**. Primers used for qRT-PCR.

## Data Availability

All data generated or analyzed during this study are included in this published article, its supplementary information files, and publicly available repositories. All RNA-seq data used for the analysis in this study are available in BnIR (http://yanglab.hzau.edu.cn/). The RNA-seq data have also been submitted to NCBI under BioProject ID: PRJNA722877 in our previous study [28]. The code used to generate the results has been uploaded to GitHub (https://github.com/liudxgit/rapeseed-develop).

## Abbreviations

DAF: Days after flowering
TAG: Triacylglycerol
ER: Endoplasmic reticulum
FAs: Fatty acids
PAs: Procyanidins
MBW: MYB, bHLH, and WD40 ternary protein complexes
SOC: Seed oil content
SCC: Seed coat content
TPM: Transcripts per million
PCA: Principal component analysis
WGCNA: Weighted gene co-expression network analysis
TWAS: Transcriptome-wide association study
TFBS: Transcription factor binding sites
MFR: Metabolic flux redirection
