# Supplementary material for: Interaction between phenylpropane metabolism and oil accumulation in the developing seed of Brassica napus revealed by high temporal-resolution transcriptomes
Source: BMC Biol. 2023 Sep 29;21:202. doi: 10.1186/s12915-023-01705-z (PMC10543336; doi:10.1186/s12915-023-01705-z)
Supplement: Supplementary file 1 — Additional file 1: Fig. S1. RNA-seq data analysis process and the core module of database. Fig. S2. Correlation between biological replications, four materials were randomly selected from the seed (Average R=0.97). Fig. S3. The correlation and PCA analysis across the transcriptomes of the 26 time points of seeds. Fig. S4. Expression patterns of seed-specific genes in all seed and non-seed samples. Fig. S5. Expression profile of marker genes in different stages. Fig. S6. WGCNA co-expression networks. Fig. S7. Enrichment analysis for genes in five core modules. Fig. S8. The co-expression network of TT5 (a), BAN (b), TT19 (c). Fig. S9. Correlation between SOC and expression of TT5, BAN and TT19. Fig. S10. Variation in the protein sequence of different haplotypes of BnaC08.TT5 (a) and BnaA08.ACLA-3 (b). Fig. S11. Correlations between the paralogues of LEC2 and LEC1. Fig. S12. Correlation between SOC and expression of LEC2, LEC1 and WRI1. Fig. S13. qPCR validation of FAs biosynthesis related genes. Fig. S14. Haplotypes for the gene ZAT4 (BnaA04G0285000ZS). Fig. S15. Variation in the protein sequence of different haplotypes of BnaA03.DOF4.4. Fig. S16. Haplotypes for the gene DOF4.7 (BnaC01G0012600ZS). Fig. S17. Variation in the protein sequence of different haplotypes of BnaC07.MORC7 (a) and BnaC01.PGI1 (b). Fig. S18. Expression profile of candidate genes. [file 12915_2023_1705_MOESM1_ESM.pdf]

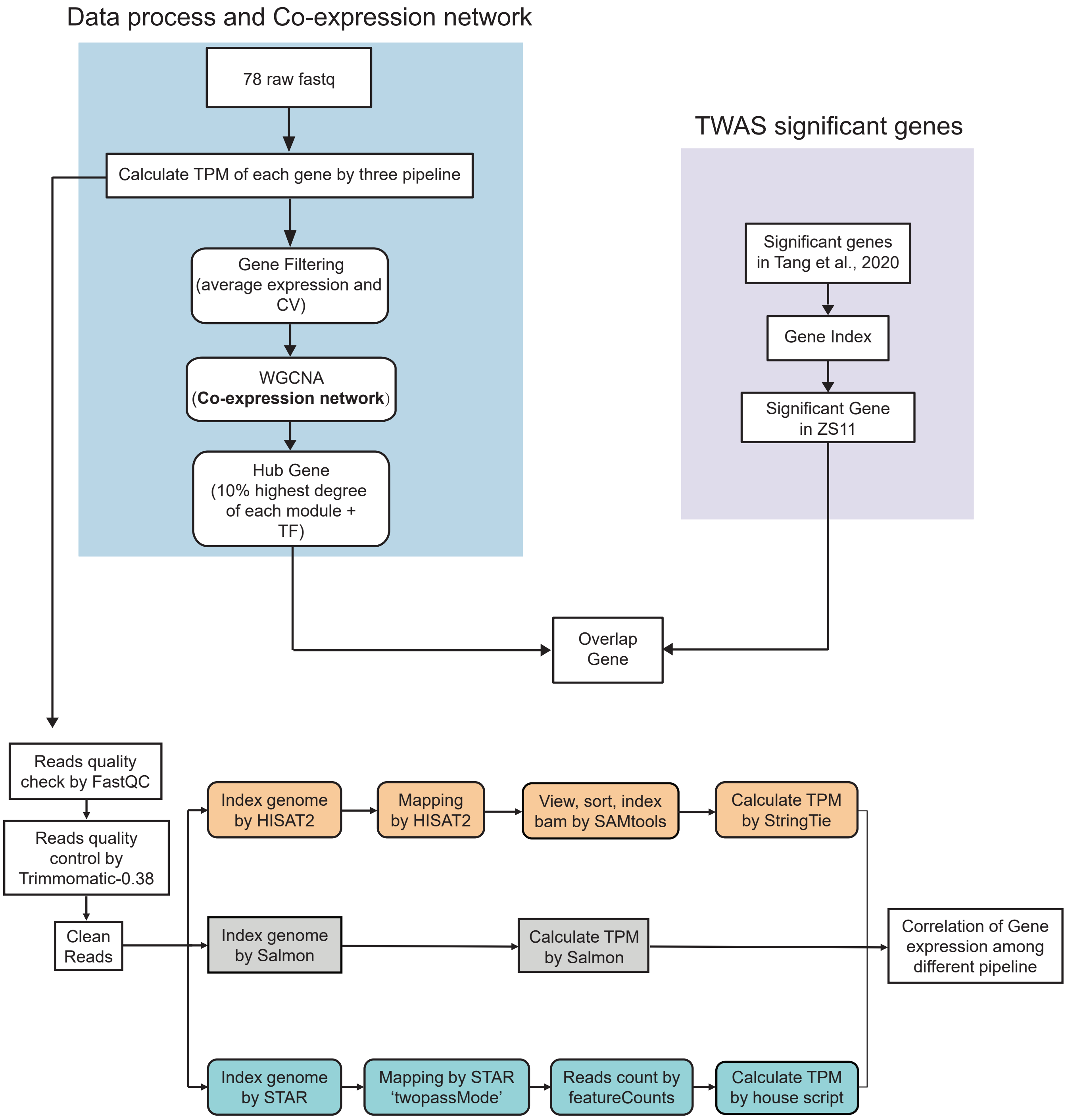

Figure S1. RNA-seq data analysis process and the core module of database.

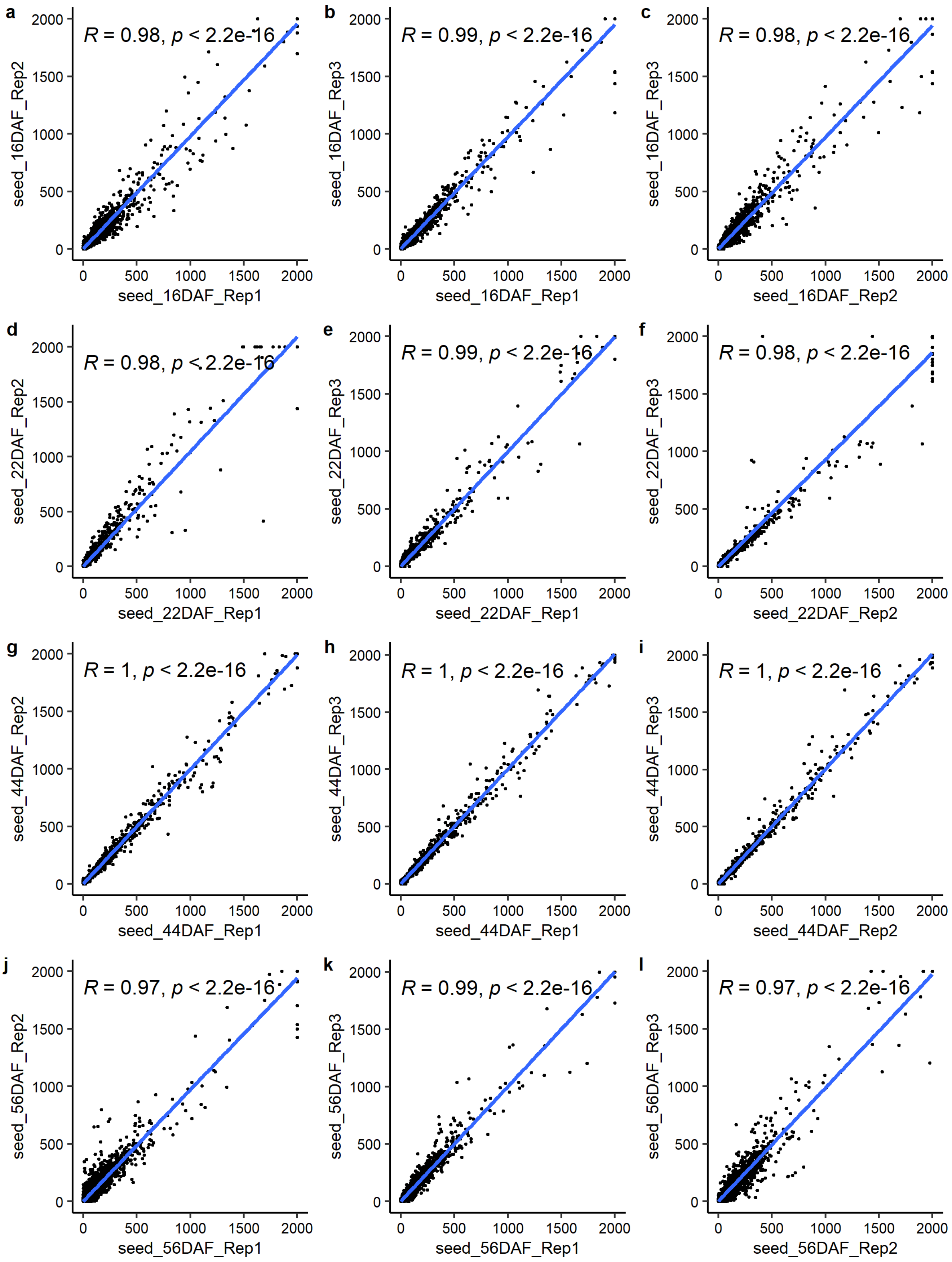

Figure S2. Correlation between biological replications, four materials were randomly selected from the seed (Average R=0.97).

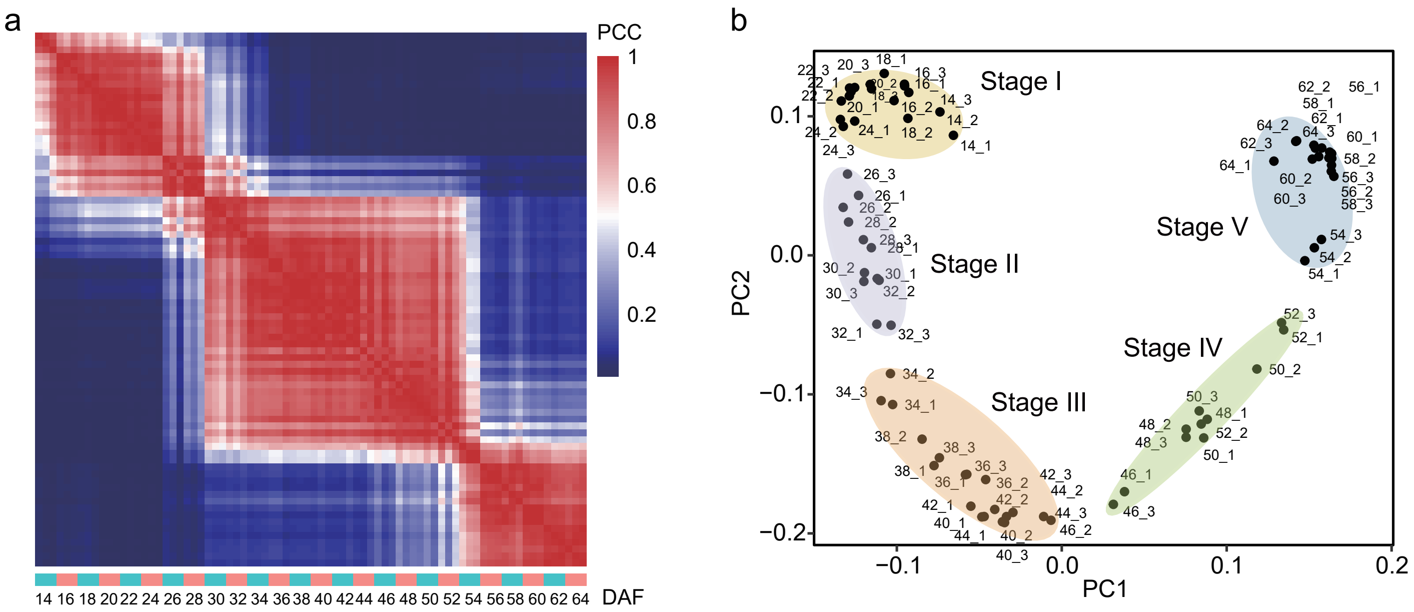

Figure S3. The correlation and PCA analysis across the transcriptomes of the 26 time points of seeds. a The heatmap of Pearson correlation coefficients across the transcriptomes of the 26 time point of seeds. b PCA of the transcriptomes of the 26 time point of seeds. Each point represents one biological replicate of each time points of seeds, and the five different colors show five development stages.

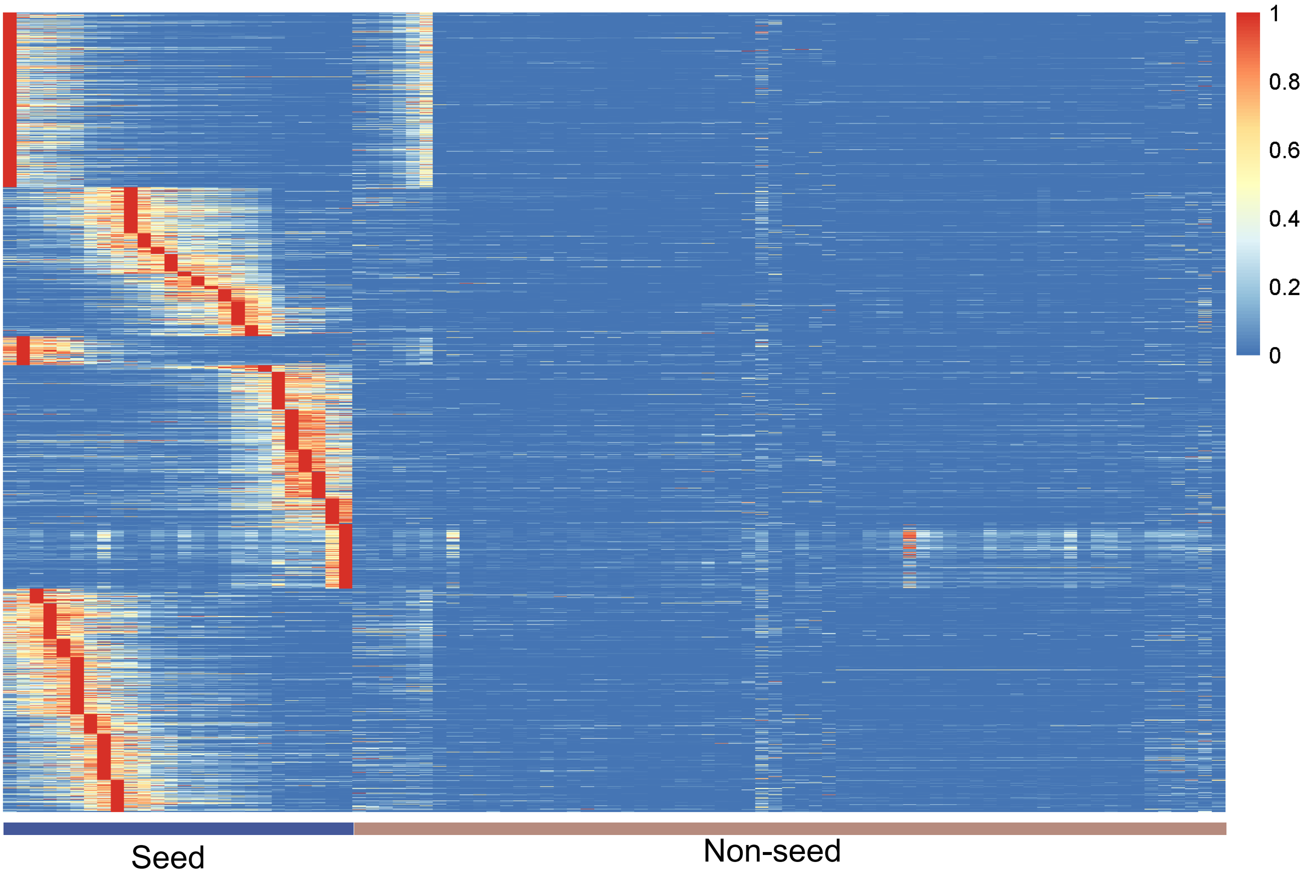

Figure S4. Expression patterns of seed-specific genes in all seed and non-seed samples. Heat map based on the TPM value normalized by the maximum value of all TPM values of the gene over all the samples used for analysis.

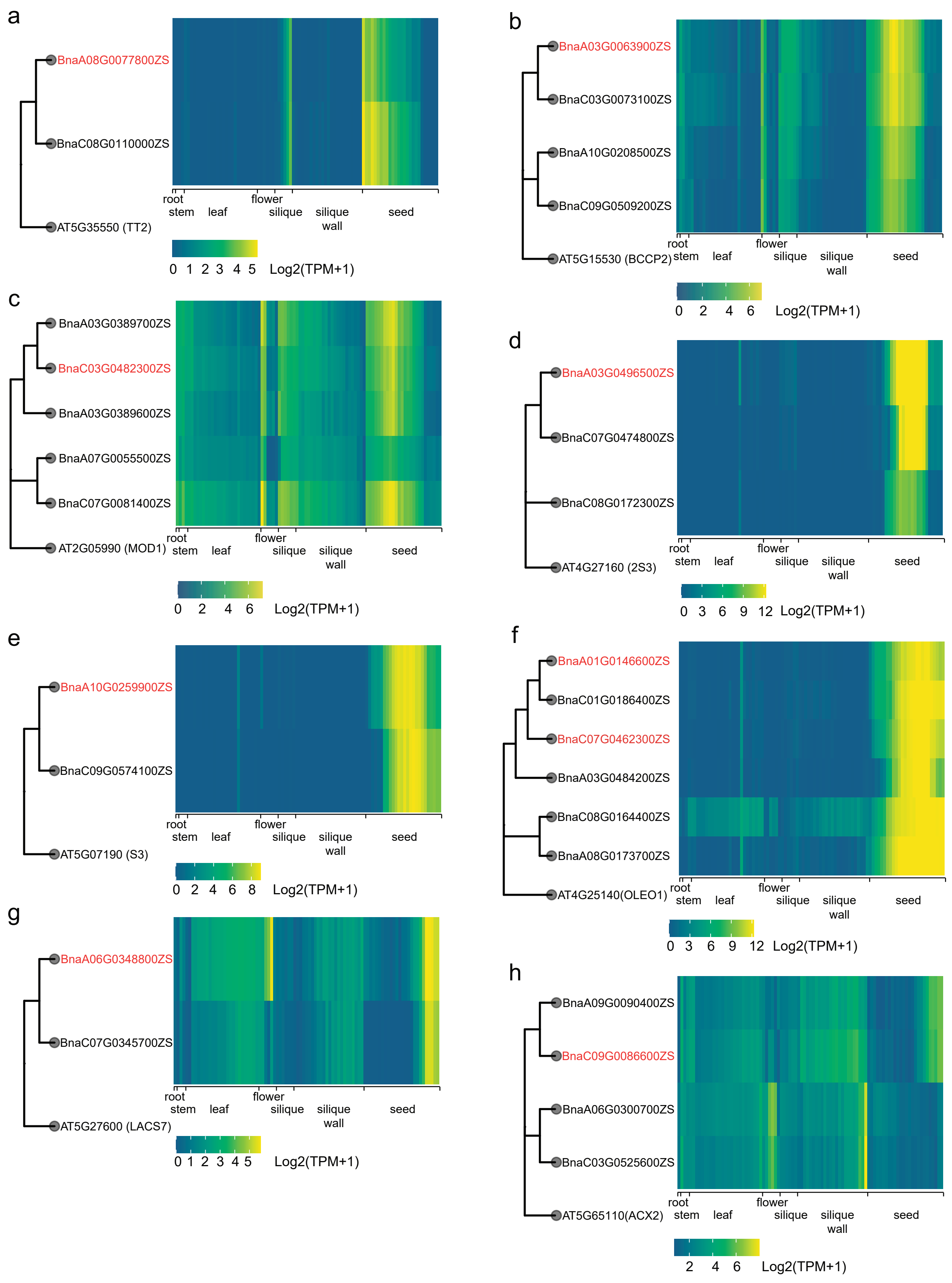

Figure S5. Expression profile of marker genes in different stages. a, The phylogenetic tree and expression profile of homologous genes of *TT2* in stage I. b, The phylogenetic tree and expression profile of homologous genes of *BCCP2* in stage II. c, The phylogenetic tree and expression profile of homologous genes of *MOD1* in stage II. d, The phylogenetic tree and expression profile of homologous genes of *2S3* in stage III. e, The phylogenetic tree and expression profile of homologous genes of *S3* in stage III. f, The phylogenetic tree and expression profile of homologous genes of *OLEO1* in stage IV. g, The phylogenetic tree and expression profile of homologous genes of *LACS7* in stage V. h, The phylogenetic tree and expression profile of homologous genes of *ACX2* in stage V.

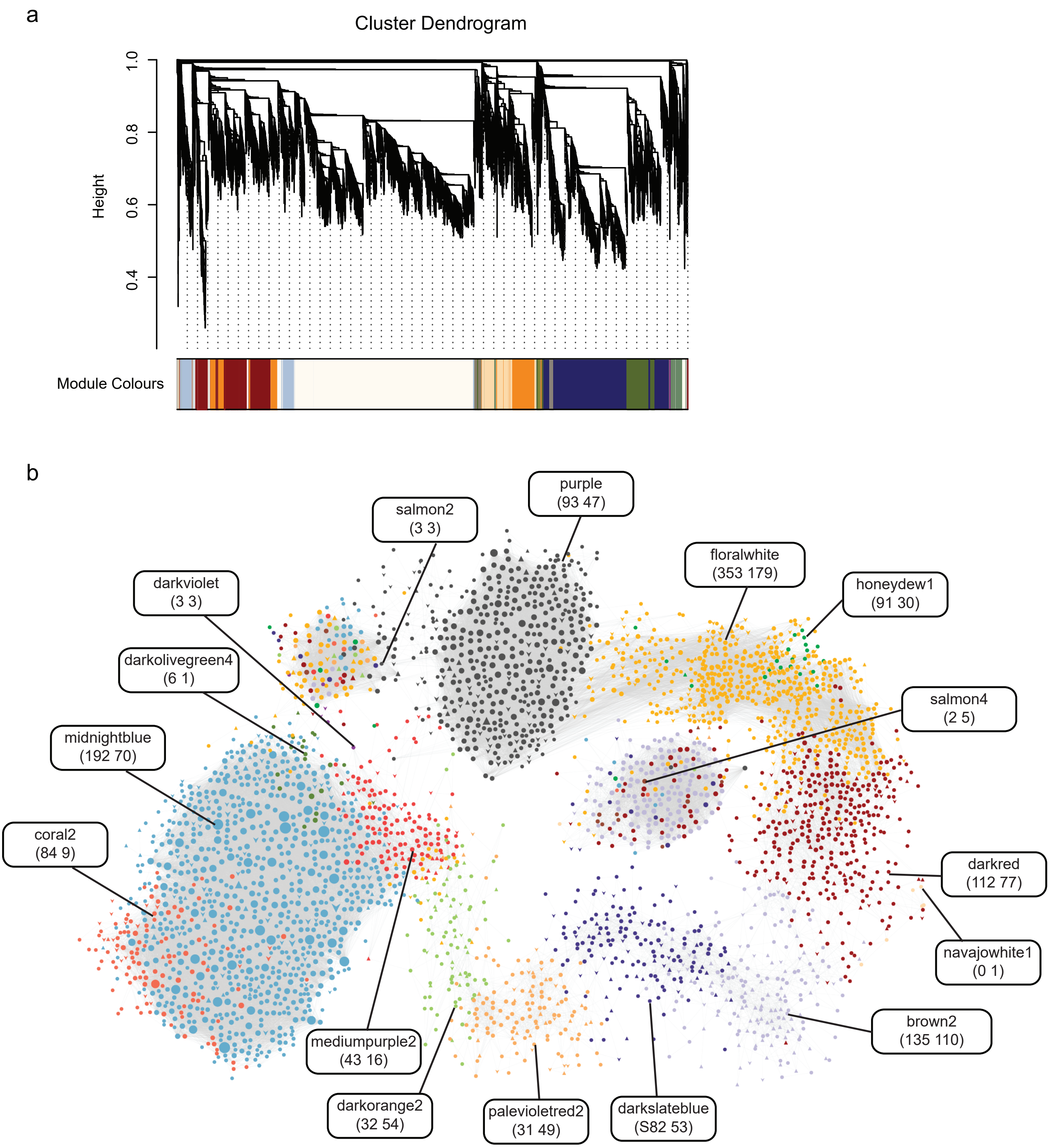

Figure S6. WGCNA co-expression networks. a, Genes cluster dendrogram of seed transcriptome. 17 modules were bult based on gene expression value. Each color indicates a different gene module. **b,** Acyl-lipid related co-expression network. The module name is shown in the picture, and the number of TFs and acyl-lipid related genes are indicated successively in brackets.

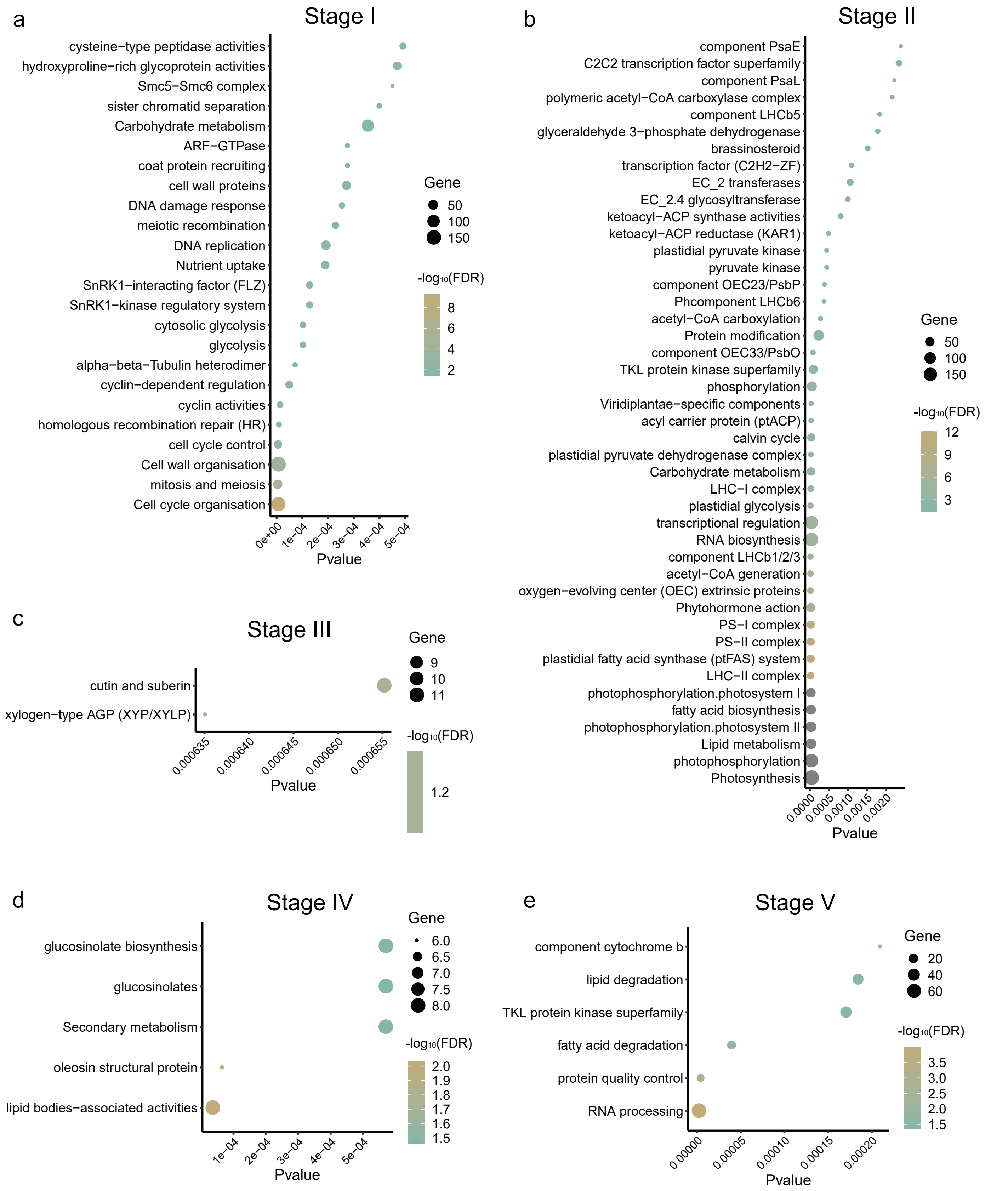

Figure S7. Enrichment analysis for genes in five core modules. a, Scatterplot of enriched pathway terms for genes in Stage I. **b,** Scatterplot of enriched pathway terms for genes in Stage II. **c,** Scatterplot of enriched pathway terms for genes in Stage III. **d,** Scatterplot of enriched pathway terms for genes in Stage IV. **e,** Scatterplot of enriched pathway terms for genes in Stage V. The size and color of the dots represent the overlapped gene number and the range of FDR values, respectively.

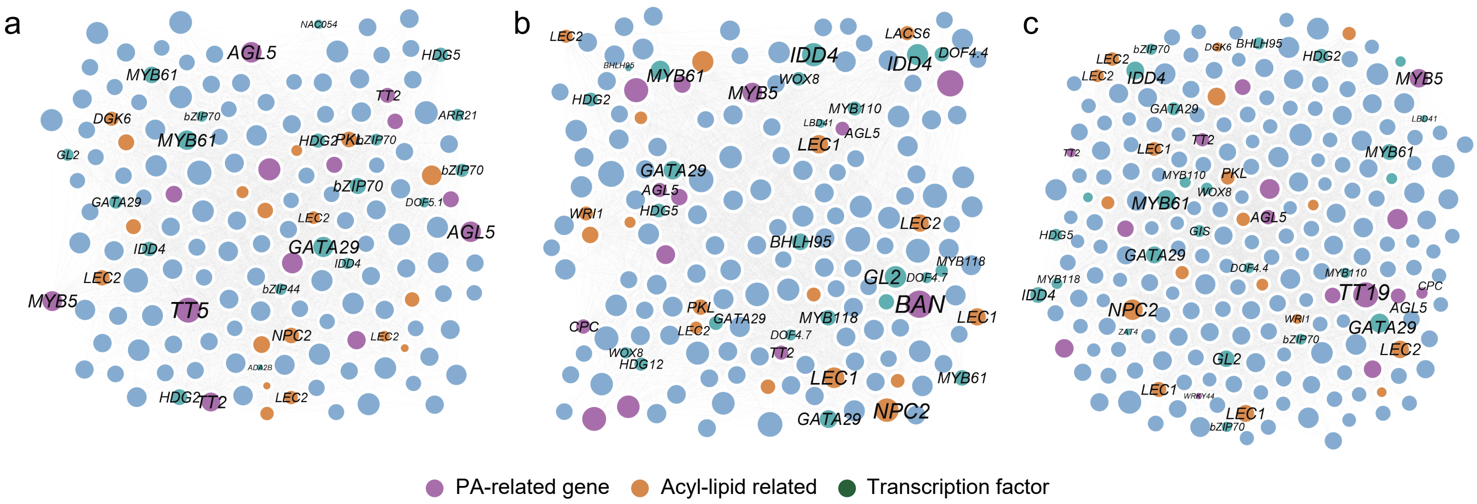

Figure S8. The co-expression network of TT5 (a), BAN (b), TT19 (c). The purple dot represented PA-related genes, the acyl-lipid related genes was represented by orange dots and the dark green dots represented transcription factor in the co-expression network.

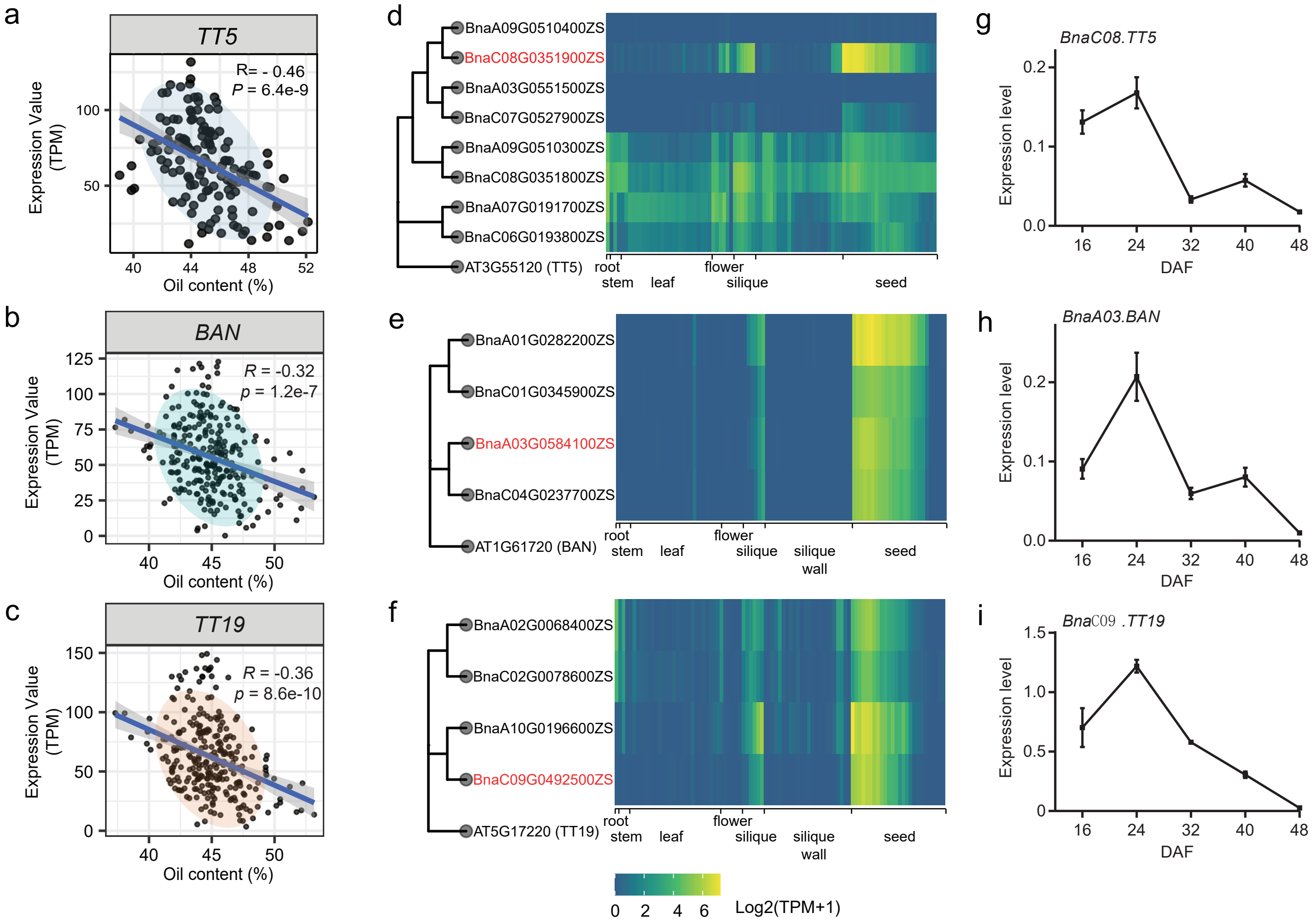

Figure S9. Correlation between SOC and expression of *TT5*, *BAN* and *TT19*. a-c Correlation between of SOC and expression of *TT5* (a), *BAN* (b) and *TT19* (c). d-f Phylogenetic tree and expression profile of *TT5* (d), *BAN* (e) and *TT19* (f). g-i qPCR validation of the expression of *TT5* (g), *BAN* (h) and *TT19* (i). *Bna.ACT7* was used as an internal control to normalize the expression level. The error bars represent the standard deviation (n = 3).

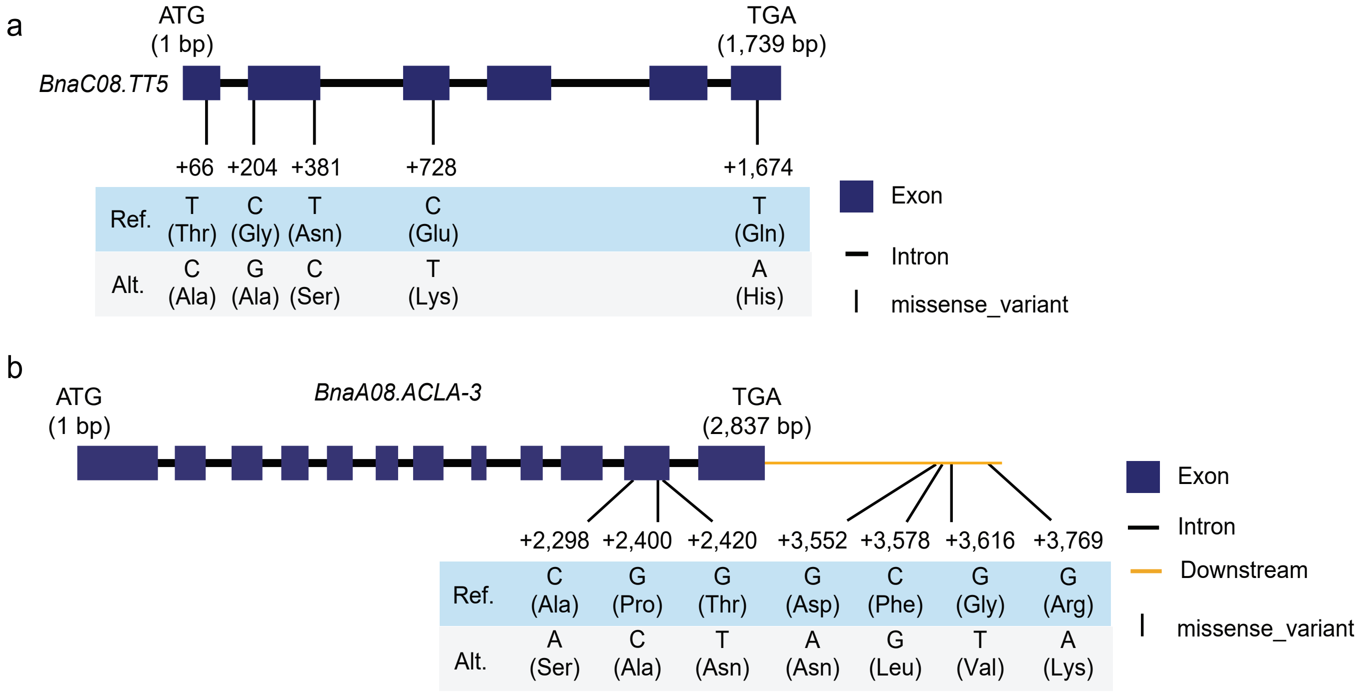

Figure S10. Variation in the protein sequence of different haplotypes of *BnaC08.TT5* (a) and *BnaA08.ACLA-3* (b).

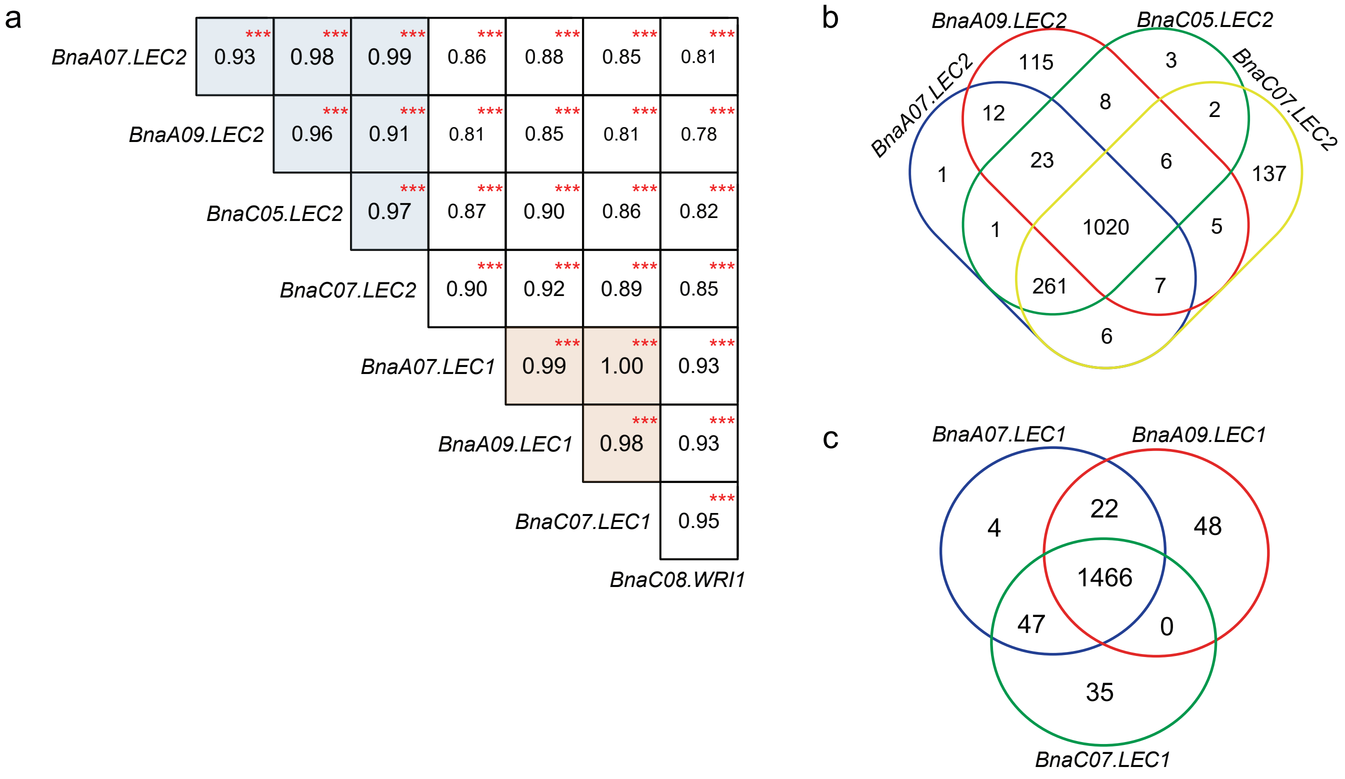

Figure S11. Correlations between the paralogues of *LEC2* and *LEC1*. a The pearson correlation coefficient of the paralogues of *LEC2*, *LEC1* and *WRI1*. b The shared number of genes in the co-expressed network of four copies of *LEC2*. c The shared number of genes in the co-expressed network of three copies of *LEC1*.

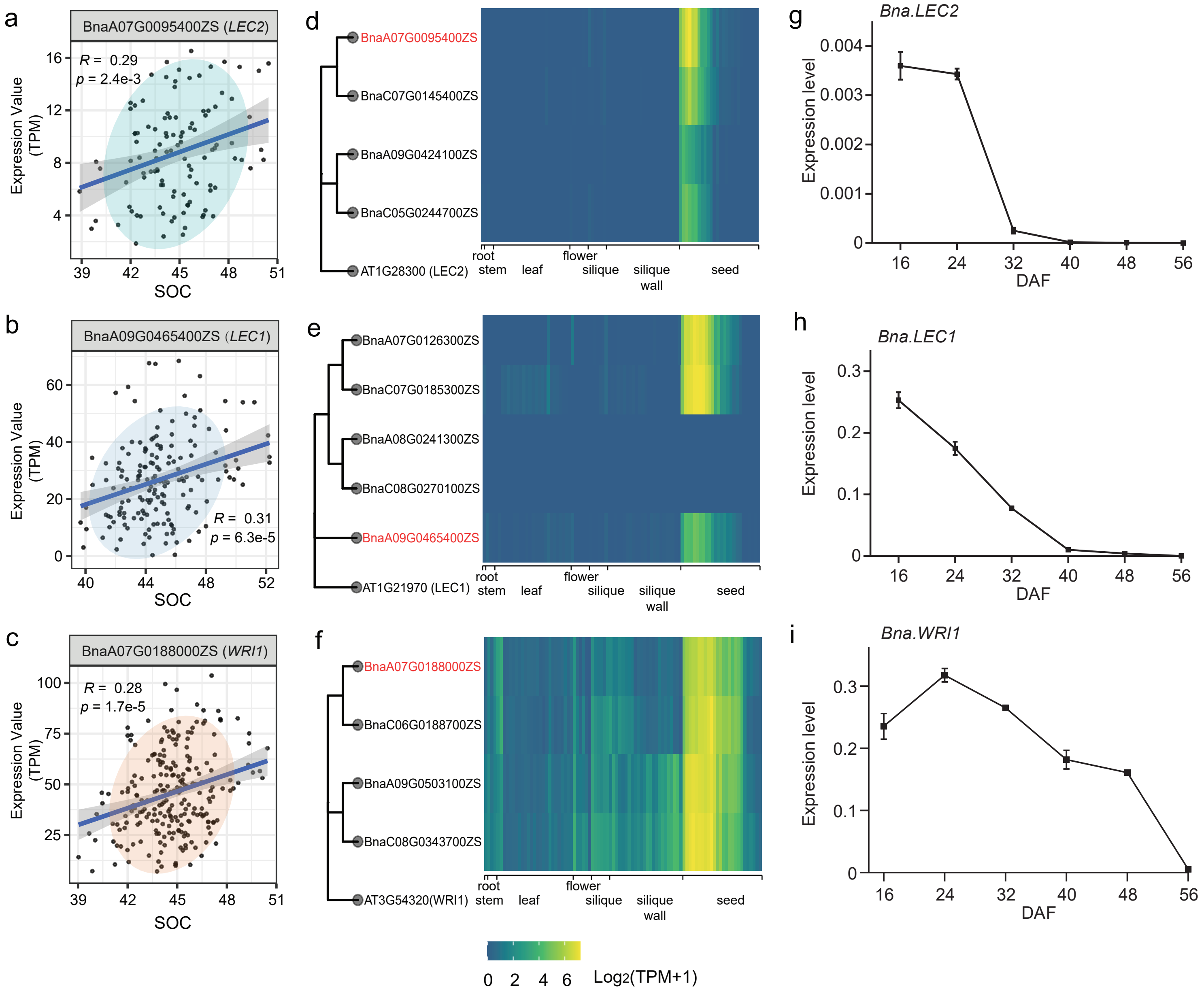

Figure S12. Correlation between SOC and expression of *LEC2*, *LEC1* and *WRI1*. a-c Correlation between of SOC and expression of *LEC2* (a), *LEC1* (b) and *WRI1* (c). d-f Phylogenetic tree and expression profile of *LEC2* (d), *LEC1* (e) and *WRI1* (f). g-i qPCR validation of the expression of *LEC2* (g), *LEC1* (h) and *WRI1* (i). *Bna.ACT7* was used as an internal control to normalize the expression level. The error bars represent the standard deviation (n = 3).

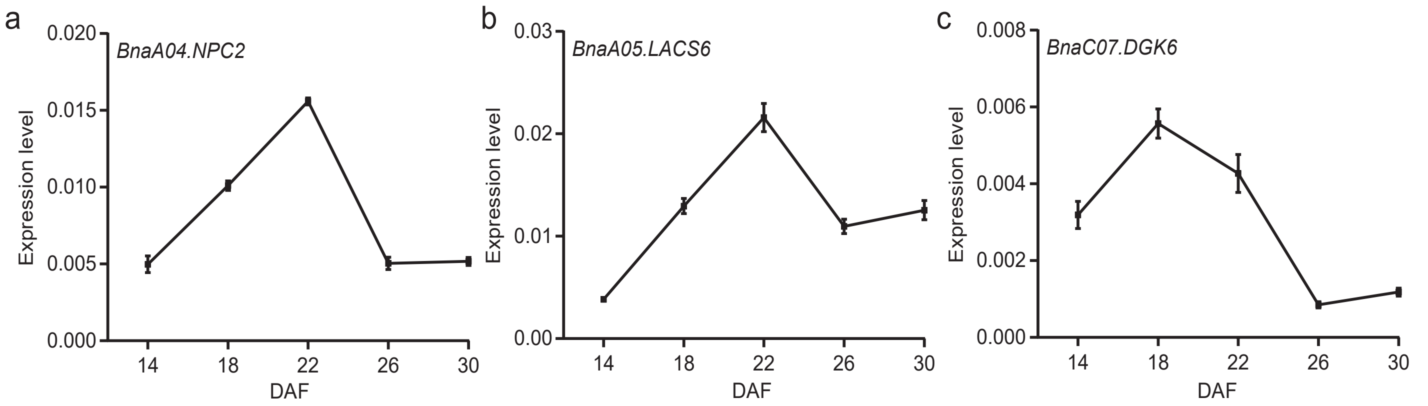

Figure S13. qPCR validation of FA biosynthesis related genes. a-c, qPCR validation of *BnaA04.NPC2* (**a**), *BnaA05.LACS6* (**b**), and *BnaC07.DGK6* (**c**). The *Bna.ACT7* gene was used as an internal control to normalize the expression level. The bars represent the standard deviation (n=3).

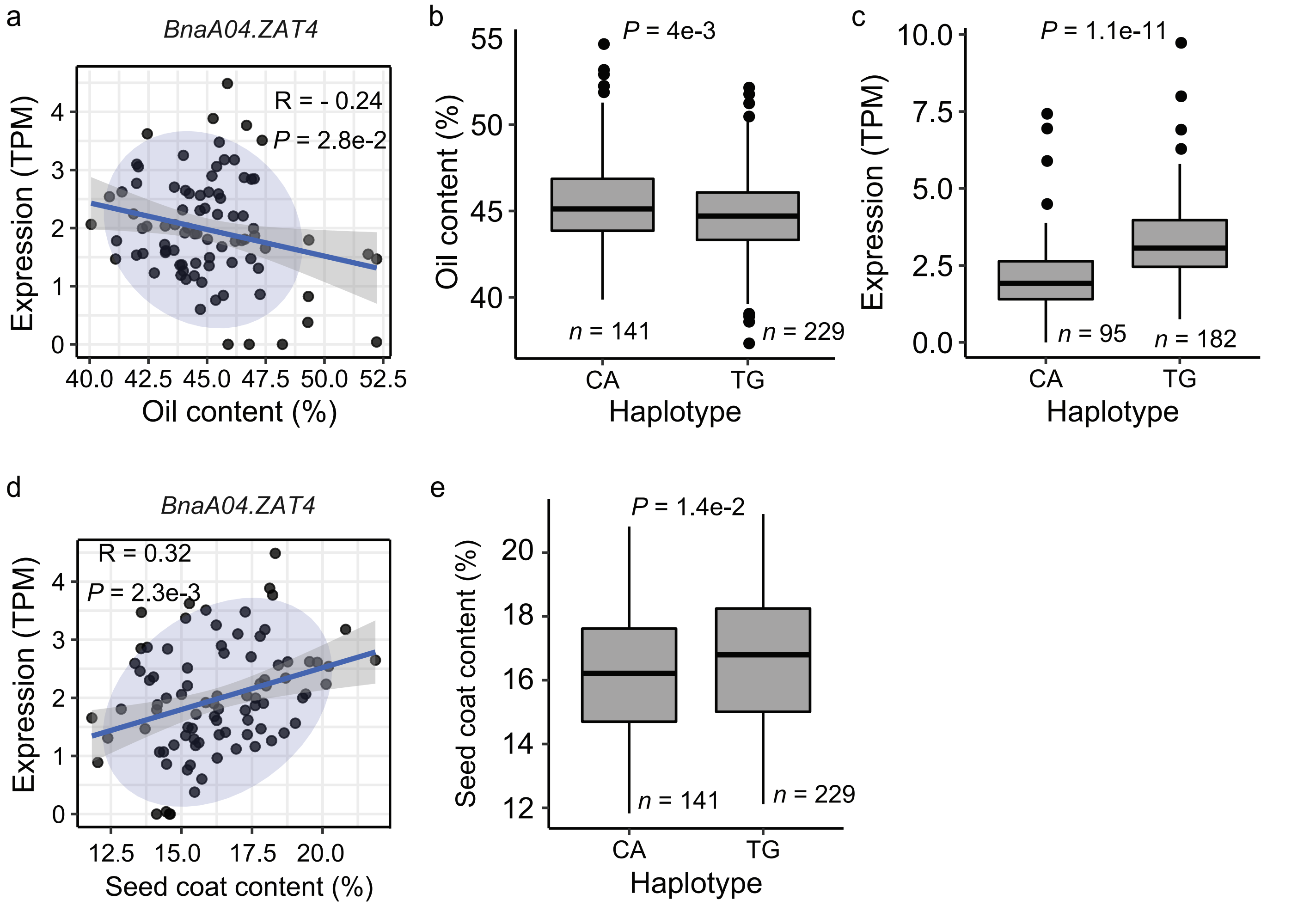

Figure S14. Haplotypes for the gene *ZAT4* (BnaA04G0285000ZS). Correlation between expression (TPM) of *BnaA04.ZAT4* with oil content (a) and seed coat content (d). Box plots for oil content (b) and seed coat content (e) for the two haplotypes (n = 141 versus 229). c Box plots for expressions for the two haplotypes (n = 95 versus 182). Center line, median; box limits, upper and lower quartiles; whiskers, 1.5× the interquartile range; dots, outliers (*P* < 2e-16, Student's t test).

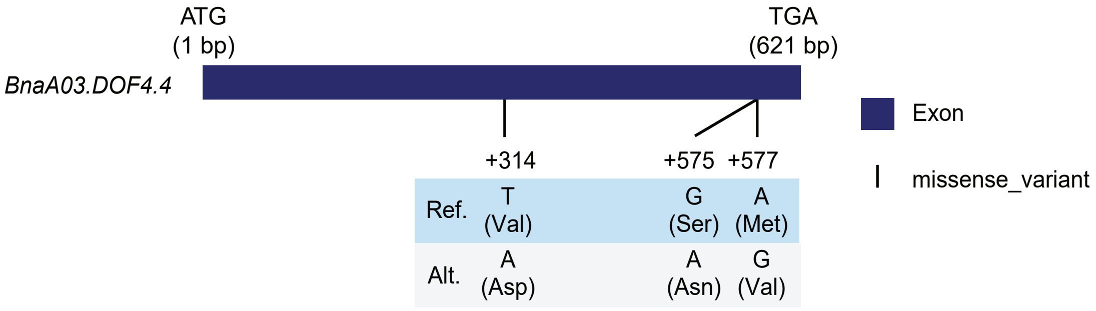

Figure S15. Variation in the protein sequence of different haplotypes of *BnaA03.DOF4.4*.

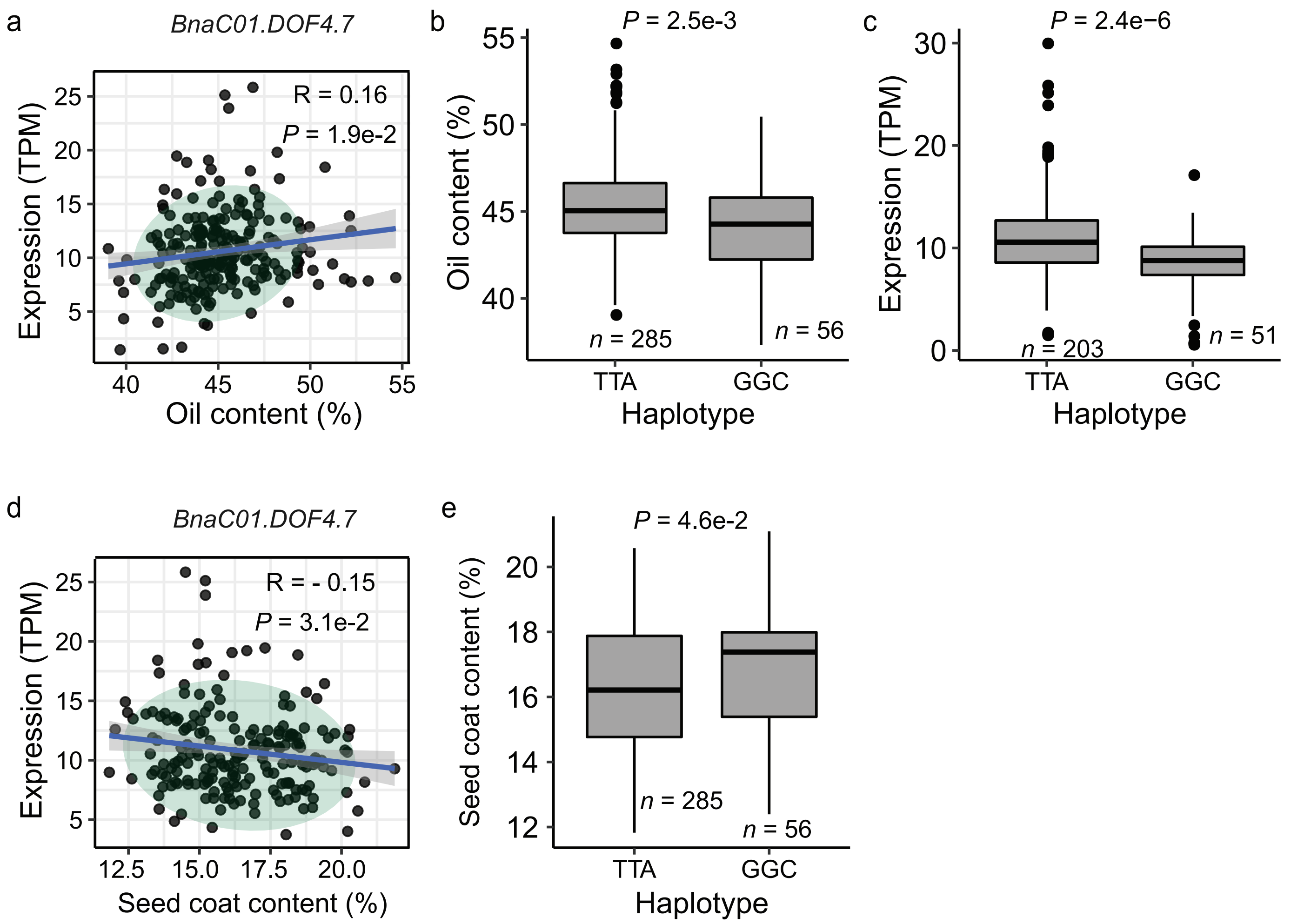

Figure S16. Haplotypes for the gene *DOF4.7* (BnaC01G0012600ZS). Correlation between expression (TPM) of *BnaC01.DOF4.7* with oil content (a) and seed coat content (d). Box plots for oil content (b) and seed coat content (e) for the two haplotypes (n = 285 versus 56). (c) Box plots for expressions for the two haplotypes (n = 203 versus 51). Center line, median; box limits, upper and lower quartiles; whiskers, 1.5× the interquartile range; dots,outliers (*P* < 2e-16, Student's t test).

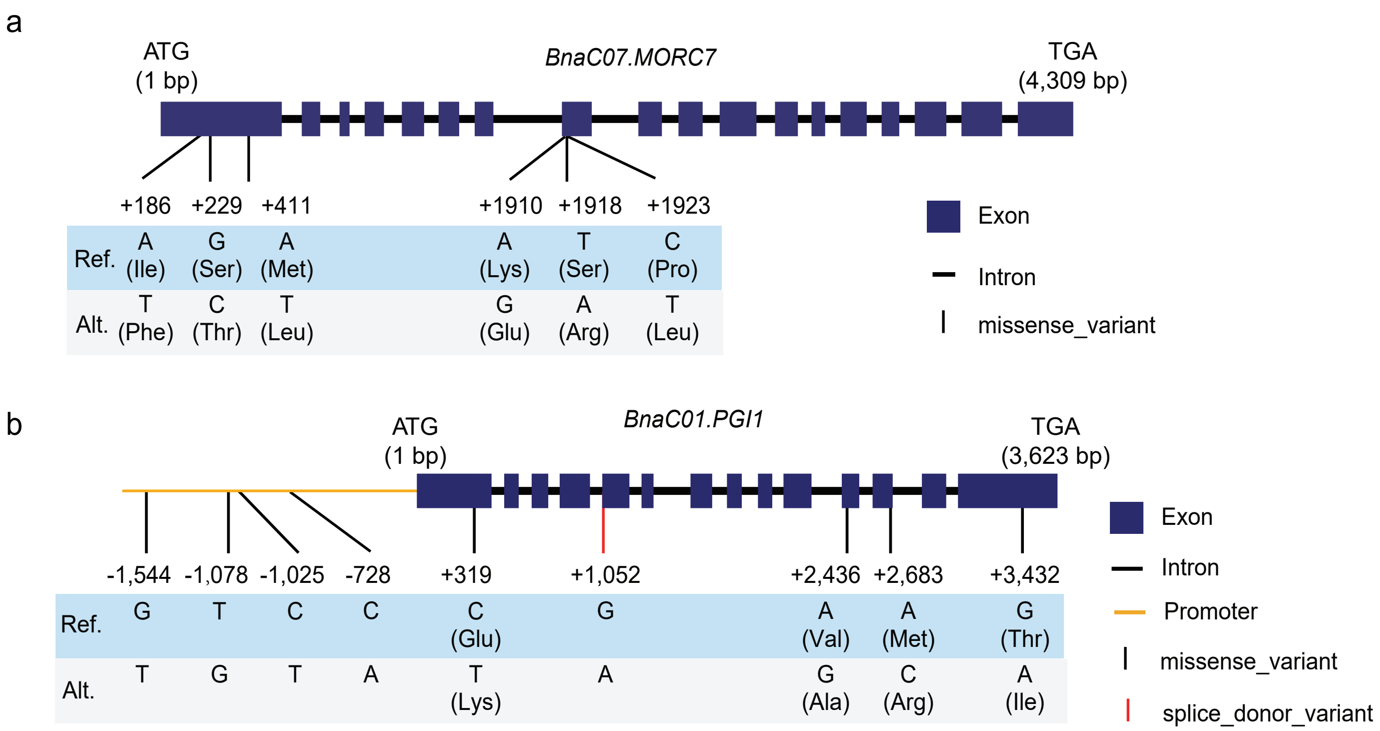

Figure S17. Variation in the protein sequence of different haplotypes of *BnaC07.MORC7* (a) and *BnaC01.PGI1* (b).

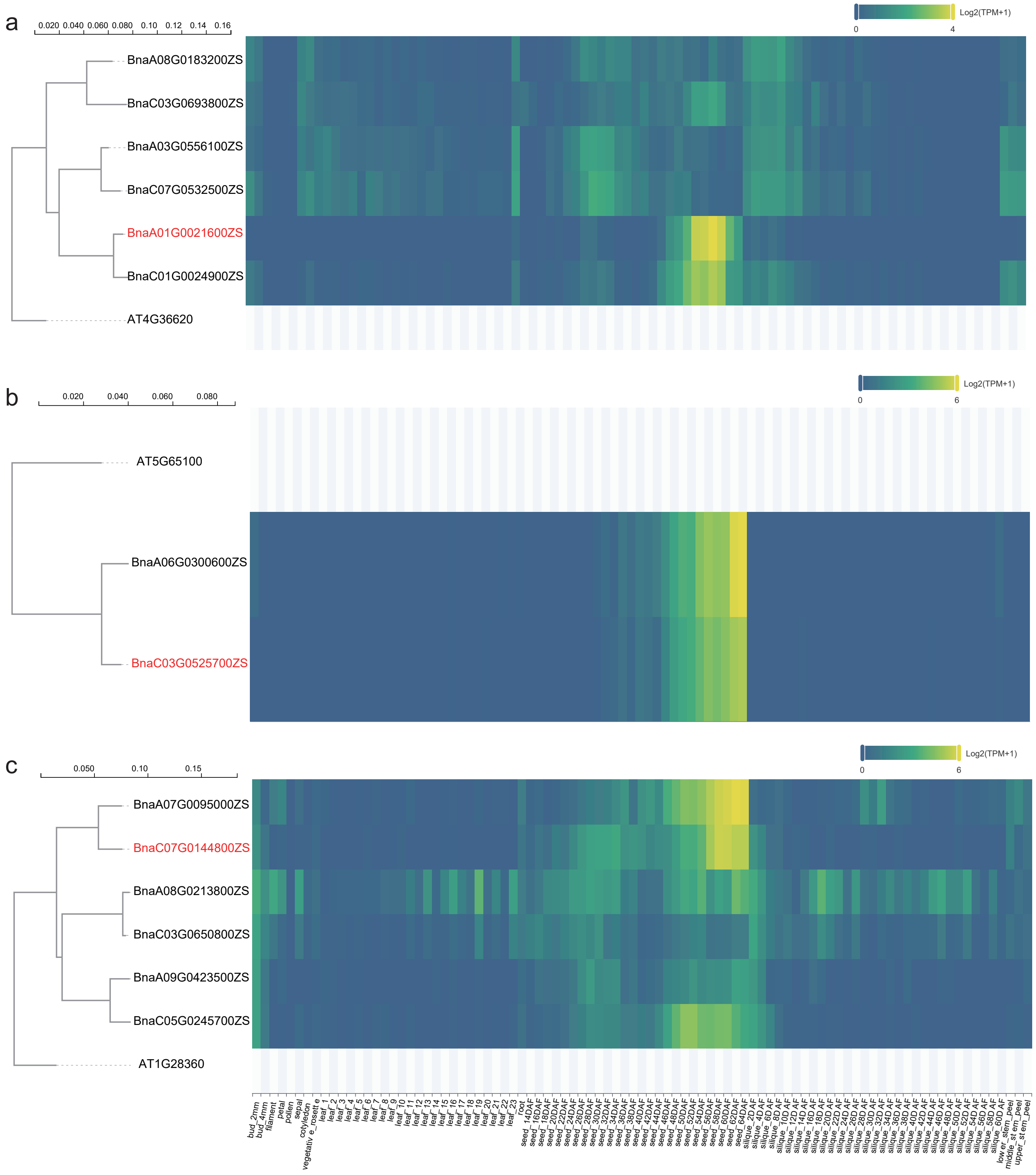

Figure S18. Phylogenetic tree and expression profile of *GATA19* (a), *EIL5* (b) and *ERF12* (c).
